# Tracheoesophageal Voicing Following Resistance‐Based Dysphagia Rehabilitation: An Exploratory Multidimensional Assessment

**DOI:** 10.1002/hed.28136

**Published:** 2025-03-25

**Authors:** Marise Neijman, Klaske E. van Sluis, Rob J. J. H. van Son, Martijn M. Stuiver, Frans J. M. Hilgers, Michiel W. M. van den Brekel, Maarten J. A. van Alphen, Lisette van der Molen

**Affiliations:** ^1^ Department of Head and Neck Oncology and Surgery The Netherlands Cancer Institute Amsterdam the Netherlands; ^2^ Amsterdam Center for Language and Communication (ACLC) University of Amsterdam (UvA) Amsterdam the Netherlands; ^3^ Center for Quality of Life and Division of Psychosocial Research and Epidemiology The Netherlands Cancer Institute Amsterdam the Netherlands

**Keywords:** high‐resolution (impedance) manometry, swallowing rehabilitation, total laryngectomy, tracheal pressure, tracheoesophageal voice

## Abstract

**Background:**

This exploratory study investigated tracheoesophageal voicing following 6 weeks of resistance‐based dysphagia rehabilitation with the SEA2.0, using a multidimensional assessment approach.

**Methods:**

Twenty laryngectomized participants were assessed at T0 (baseline), T1 (after 6 weeks training), and T2 (after 8 weeks rest). Training included the chin tuck, jaw opening, and effortful swallow against resistance. Multidimensional assessments included acoustic analysis (AVQI, intensity, dynamic range), clinician‐rated perceptual evaluations (voice tonicity, intelligibility), PROMs (VHI‐10, V‐RQOL, CPIB‐10), and tracheal manometry and pharyngeal‐/esophageal high‐resolution impedance manometry during voicing.

**Results:**

No significant changes in mean AVQI, intensity, dynamic range, voice tonicity, intelligibility, PROMs, and physical outcomes (manometry) were found. However, several clinically relevant changes, mostly improvements, were noted in 14 participants for AVQI and PROMs.

**Conclusion:**

Tracheoesophageal voice, on average, is not affected by resistance‐based muscle training for swallowing rehabilitation. For some participants, however, several clinically relevant improvements in voice quality and quality of life were noticeable.

## 1 Introduction

Total laryngectomy results in impairments of several vital functions (i.e., speaking, breathing, smelling, and swallowing) [1, 2, 3]. However, over the past decades, it has become possible to diminish many of these impairments due to advances in post‐laryngectomy rehabilitation (e.g., heat and moisture exchangers [HMEs] significantly can diminish pulmonary function impairment, the polite yawning technique [a nasal airflow‐inducing maneuver] can restore the sense of smell in many patients, and most recently, resistance‐based exercises can improve swallowing) [4, 5, 6, 7, 8, 9]. With respect to voice rehabilitation, besides the more traditional methods of esophageal and electro‐larynx voice, tracheoesophageal voice prostheses have become the preferred rehabilitation option [5, 10].

Voice prostheses are one‐way valves positioned in a tracheoesophageal fistula, enabling the patient, by occluding the stoma, to direct pulmonary air into the pharyngo‐esophageal segment/neopharynx. This air brings the mucosa there into vibration and produces sound for speech. The pharyngo‐esophageal segment thus serves as the new sound source [11]. Patients now regain pulmonary‐driven speech, but in some cases also enable singing and/or whispering [12, 13]. Tracheoesophageal voice and speech are not guaranteed optimal, though, as voice quality, intelligibility, and experienced voice handicap vary considerably between patients [10].

Assessing tracheoesophageal voice requires a multidimensional assessment approach, using both subjective and objective voice parameters [14, 15]. Subjective parameters of tracheoesophageal voice quality include clinician‐ and patient‐rated perceptual evaluation of voice and speech, and patient‐reported outcome measurements (PROMs) [10]. Perceptual evaluation by trained clinicians is presently the gold standard for assessing voice quality and speech intelligibility. One of the rated characteristics is the tonicity of the voice, that is, hypertonic, normotonic, or hypotonic, describing how strained or weak the voice sounds. This tonicity depends on the condition of the musculature and the connective tissues in the PE‐segment, being the sound‐producing element in tracheoesophageal speech [16]. The tonicity of the PE‐segment muscles can be influenced by several factors, such as whether the musculature was denervated or myotomized, the type of reconstruction and the shape and size of the segment closure, and the impact of postradiation fibrosis [17, 18].

Examples of objective parameters are intensity (in dB), dynamic range (in dB), pitch (in Hz), harmonic‐to‐noise ratio, perturbation, breathiness, and the overall Acoustic Voice Quality Index (AVQI). The AVQI is a widely used measure that reflects several acoustic outcomes within a single scoring system [19, 20]. Although the AVQI is reliable and valid for laryngeal voices, it is not (yet) validated for tracheoesophageal voices. Additional objective parameters relevant for voice quality assessment could come from tracheal and pharyngeal (high‐resolution impedance) manometry.

**FIGURE 1:**
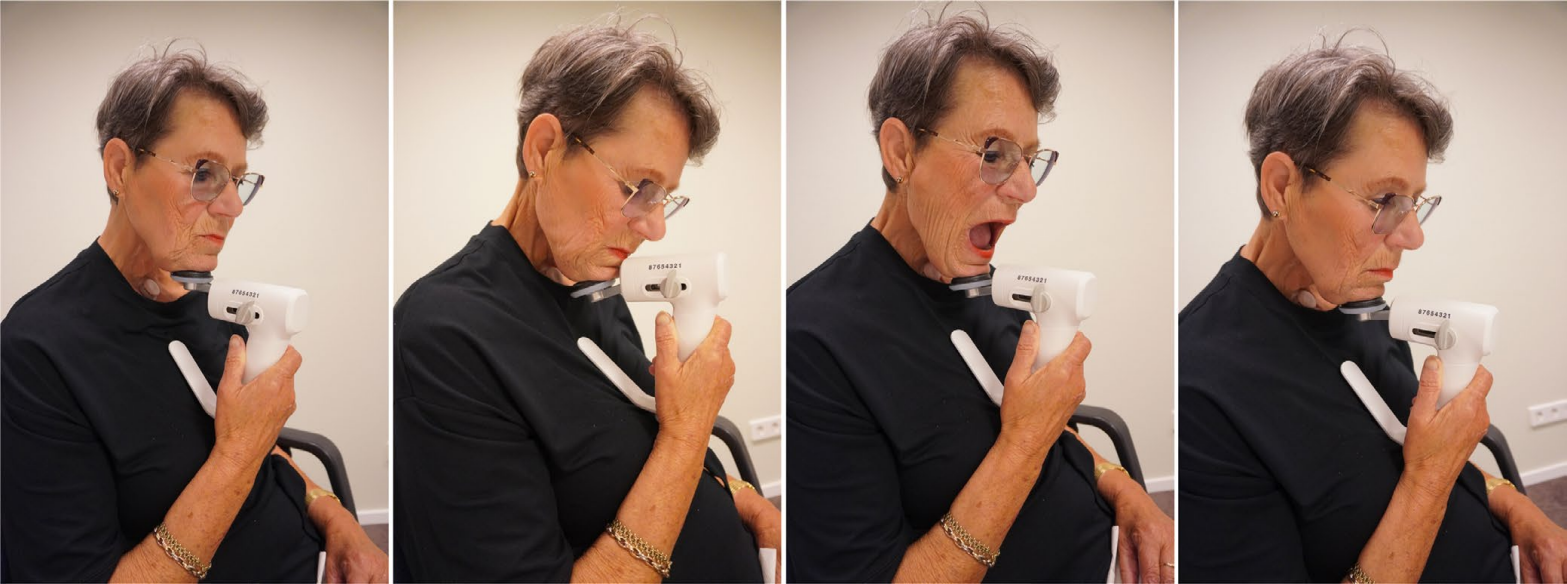
Exercises performed with the Swallowing Exercise Aid 2.0 (SEA2.0). From left to right: (1) resting position, (2) chin tuck against resistance, (3) jaw opening against resistance, (4) effortful swallow against resistance. Picture of the Swallowing Exercise Aid 2.0 (SEA2.0). [Color figure can be viewed at wileyonlinelibrary.com]

In patients who have undergone a total laryngectomy, the functions of swallowing and voicing are closely connected, as they rely on the same anatomical structures. This means that pressure buildup is essential not only for producing voice but also for effective swallowing. Although medical treatments for swallowing issues, such as dilatation, are sometimes necessary, negative effects on tracheoesophageal voice quality are often observed in clinical practice, likely due to their impact on the PE segment. Recently, we conducted a Clinical Phase II rehabilitation trial with laryngectomized participants who had self‐reported (and objectively confirmed) dysphagia, 45% of whom also experienced voice difficulties [8]. In the Clinical Phase II trial, participants underwent an intensive resistance‐based swallowing therapy program using the novel Swallowing Exercise Aid 2.0 (SEA2.0) (see Figure 1) [8, 9]. This handheld device enabled them to perform swallowing exercises against adjustable resistance; one of the principles of strength training [8, 9, 21]. The therapy program included chin tuck against resistance (CTAR), jaw opening against resistance (JOAR), and effortful swallow against resistance (ESAR), performed three times daily over 6 weeks. The CTAR and JOAR exercises involved 30 isokinetic repetitions and three isometric holds of 60 s each, whereas the ESAR exercise included 10 repetitions [8]. The trial demonstrated positive outcomes for swallowing. After 6 weeks of training, participants showed significant improvements in muscle strength, swallowing capacity, swallowing efficiency, and patient‐reported swallowing and quality of life. These gains persisted after an 8‐week rest period and remained evident 6–7 months later [9].

Given the close relationship between swallowing and voicing, it was deemed relevant to also evaluate the possible effects of this adjustable resistance‐based rehabilitation program on voicing. Improvements in one function may not necessarily benefit the other. Conversely, when considering the neuroplasticity phenomenon, one may even expect some improvements. For instance, research in Parkinson's disease with the Lee Silverman Voice Treatment (LSVT) training showed significant improvements not only in voice quality but also in swallowing [22]. Therefore, this exploratory study aimed to investigate tracheoesophageal voicing following a resistance‐based dysphagia rehabilitation program, using a multidimensional evaluation approach.

## 2 Materials and Methods

This study analyzed data obtained in a Clinical Phase II trial that was approved by the Medical Ethical Committee of the Netherlands Cancer Institute (METC21.0904/N21STL) [8]. The guidelines of the Helsinki Declaration were followed, and written informed consent was obtained from each participant before inclusion. Data were collected at the Head and Neck Oncology Department of a tertiary National Cancer Institute.

### 2.1 Participants

Between April 2022 and February 2023, laryngectomized individuals with self‐reported dysphagia were recruited from the Netherlands Cancer Institute and via the Dutch Patient Association for Head and Neck (PVHH). In total, 20 participants (15 males) with a median age of 71 years (range 45–78) at baseline were included. All 20 participants completed the baseline (T0) and short‐term (T1) assessments after 6 weeks of training. One participant (S12) was excluded after the T1 assessments because of health issues (not related to the rehabilitation treatment), leaving 19 participants for the long‐term (T2) assessments after 8 weeks of rest. Table 1 shows the characteristics of the participants.

**TABLE 1:** Participant characteristics (previously published [8] and information about tracheoesophageal voice added).

Participant			Tumor		Total laryngectomy						Rehabilitation		
	Sex	Age	Location	TNM	Indication	Post‐TL (months)	Pharynx closure	Myotomy	Stenosis (dilatations)	Timing (C)RT	Voice prosthesis	Stoma appliance	Airway protection
S01	M	78	Hypopharynx	T1N1	Functional	47	T/Y	No	No (0)	Pre	Yes	Adhesive	HME
S02	M	65	Hypopharynx	pT4aN0	Curative	73	PM	Yes	Yes (2)	Post	Yes	Adhesive	HME
S03	M	75	Larynx	cT2NM	Salvage	75	T/Y	Yes	Yes (1)	Pre	Yes	Larytube	HME
S04	M	73	Hypopharynx	cT3N2b	Curative	40	PM	No	Yes (1)	Pre	Yes	Adhesive	HME
S05	F	62	Trachea	pT4bN0	Curative	35	Horizontal	Yes	No (0)	Post	Yes	Adhesive	HME
S06	M	53	Larynx	pT4N2a	Curative	12	T/Y	Yes	No (0)	No	Yes	Adhesive	HME
S07	M	72	Larynx	T1bN0	Salvage	94	Vertical	Yes	No (0)	Pre	Yes	Adhesive	HME
S08	M	71	Larynx	cT2N0	Salvage	44	PM	Yes	No (0)	Pre	Yes	Adhesive	HME
S09	M	76	Hypopharynx	T3N2c	Curative	47	T/Y	Yes	Yes (2)	Post	Yes	Adhesive	HME
S10	M	61	Larynx	cT4aN0	Curative	47	T/Y	Yes	No (0)	No	Yes	Adhesive	HME
S11	M	67	Larynx	cT4aN0	Curative	65	T/Y	Yes	No (0)	No	Yes	Adhesive	HME
S12	M	77	Larynx	T4NM	Salvage	274	T/Y	Yes	No (0)	Pre	Yes	No	BIB
S14	F	45	Larynx	cT3N0	Salvage	22	T/Y	Yes	Yes (2)	Pre	Yes	Adhesive	HME
S15	M	50	Hypopharynx	pT4N3b	Curative	33	SCAIF	No	No (0)	Post	Yes	Adhesive	HME
S16	M	66	Larynx	pT4aN0	Curative	16	T/Y	Yes	No (0)	Post	Yes	Adhesive	HME
S17	M	63	Larynx	T2N0	Salvage	67	Vertical	Yes	No (0)	No	Yes	Adhesive	HME
S18	M	70	Larynx	rT2N0	Salvage	9	T/Y	No	No (0)	Post	Yes	Adhesive	HME
S19	F	73	Larynx	T3N2b	Curative	147	T/Y	Yes	Yes (2)	No	Yes	Larybutton	HME
S20	M	77	Larynx	T4aN0	Salvage	138	Vertical	Yes	No (0)	Post	Yes	Larybutton	HME
S21	F	73	Hypopharynx	T4aN0	Salvage	87	Gastric pull‐up	No	No (0)	No	Yes	Adhesive	HME
Median (range)		71 (45–78)				47 (9–274)							

*Note*: Table copied from the previously published study and supplemented with data about tracheoesophageal speech rehabilitation.

Abbreviations: ALT, anterolateral thigh flap; BIB, cloth stoma cover; (C)RT, (chemo) radiation therapy; HME, heat and moisture exchanger; PM, pectoralis major flap; SCAIF, supraclavicular artery island flap; TNM, a classifying system for malignancy consisting of T (tumor) N (node), and M (metastasis).

### 2.2 Multidimensional Assessment

Objective and subjective voice outcomes were evaluated with a multidimensional assessment program as recommended in the literature [14]. Primary objective outcome measures included voice recordings to assess voice quality (AVQI), intensity and dynamic range (in dB), and subjective outcome measures included clinician‐rated perceptual evaluation of the voice tonicity and intelligibility and PROMs. Secondary physiological outcome measures were tracheal manometry and pharyngoesophageal high‐resolution impedance manometry (HRIM) during voicing.

#### 2.2.1 Voice Quality

Voice quality was assessed using recordings from the tracheal pressure measurement only, which included a route description for spontaneous continuous speech, sustained vowels/a/at neutral, soft, low, high, and loud pitches, and reading aloud three sentences along with the Dutch text “80 dappere fietsers” (80 brave cyclists). The voice recordings were recorded with the Sony ICD‐AX412F and saved as MP3 files. Three seconds of the sustained vowel, and 4 s of continuous speech were used for the acoustic analysis using the AVQI v2.03 in PRAAT version 6.2.09 [23, 24]. Segments were selected from sustained vowel and continuous speech recordings for having the highest number of voiced frames as measured with PRAAT. Although the AVQI has not been specifically validated for individuals who have undergone a laryngectomy, it was selected due to its strong correlations with perceptual assessments by speech and language pathologists (SLPs), the absence of alternative options, and its prior use in voice quality research in laryngectomized patients [5, 10]. The AVQI algorithm takes the cepstral peak prominence, harmonics‐to‐noise ratio, shimmer, and the slope and tilt of the regression line through the long‐term average spectrum into account [10]. The AVQI score ranges between 0 and 10, with lower scores defined as better voice quality. In Dutch, scores above the cut‐off score of 2.95 are indicated as distorted [10]. In non‐laryngectomized patients, a change of 0.95 points on the AVQI is considered clinically relevant [25].

The voice intensity (in dB) was measured as the average value per participant over a 3‐s segment of a sustained vowel at neutral pitches, using PRAAT software. For consistency, this 3‐s segment was the same one utilized in the AVQI analysis. Additionally, the mean intensity was calculated for each entire task individually (including neutral, soft, low, high, and loud pitches). The dynamic range (in dB) was determined for each participant by subtracting the mean intensity of the soft vowel/a/from that of the loud vowel/a/.

#### 2.2.2 Clinician‐Rated Perceptual Voice Tonicity and Intelligibility

To assess perceptual tracheoesophageal voice tonicity and intelligibility, five experienced SLPs conducted a listening experiment. In this experiment, 8 s of speech, four from spontaneous speech and four from the read‐aloud text, were evaluated using a digital five‐point adjective scale. For voice tonicity, the scale ranged from (1) *Strongly hypotonic* via (3) *Normotonic* to (5) *Strongly hypertonic*, while for intelligibility, it ranged from (1) *Very poorly intelligible* via (3) *Neither poorly nor well intelligible* to (5) *Very well intelligible* (see Appendix S1).

#### 2.2.3 PROMs

Although there are currently no specific questionnaires available for assessing voice function in laryngectomized individuals, participants' perceived subjective voice function was evaluated using various commonly used voice and speech quality questionnaires (e.g., Voice Handicap Index [VHI‐10], Voice‐Related Quality Of Life [V‐RQOL], and Communicative Participation Item Bank [CPIB‐10]).

VHI‐10 (range 0–40) was employed to assess participants' perspectives on their voice‐related difficulties. For patients with voice problems who have a larynx, the VHI‐10 is validated [26]. A higher score on the VHI‐10 indicates that the patient experiences more complaints about their voice. In non‐laryngectomized patients, a VHI‐10 score higher than 11 is considered abnormal, and a change of five points on the VHI‐10 is considered clinically relevant [26, 27, 28].

V‐RQOL (range 0–100) questionnaire was used to assess participants' V‐RQOL. The V‐RQOL consists of 10 items and was found valid and reliable for patients with an intact larynx [29]. A higher score on the V‐RQOL is considered as better quality of life. In non‐laryngectomized patients, a change of 15–20 points on the V‐RQOL is considered clinically relevant [29].

Communicative participation was assessed using the CPIB‐10 (range 0–30). The CPIB‐10 includes 10 self‐reported items that assess a disease's impact on daily conversation situations [30, 31]. Although the CPIB‐10 is not specifically designed for laryngectomized participants, laryngectomized individuals were included in the cohort during the validation process. Therefore, the CPIB‐10 is also validated for use with this population. Higher scores indicate less interference in participation.

The results for the primary outcomes between T0 versus T1, T1 versus T2, and T2 versus T0 were summarized as the mean and range of changes, and percentages of patients with improved, worsened, or equal outcomes per outcome measure. Additionally, for more detailed insight, increases, decreases, or no changes were reported per outcome measure for each participant, and color coded according to clinical relevance (see Table 3).

#### 2.2.4 Tracheal Manometry

**FIGURE 2:**
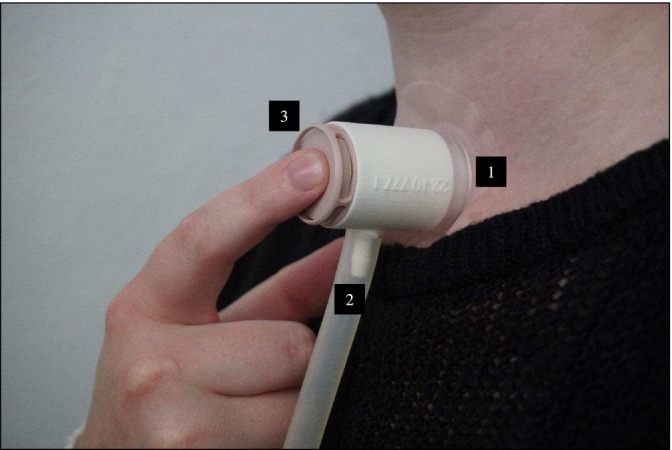
The 3D‐printed connector for the tracheal pressure measurement with the FLUKE Gasflow Analyzer VT590 consists of a ring that fits the stoma adhesive (1), a cone that accommodates the 1‐m plastic flexible tube between the connector and the GFA device (2), and a front opening for the participant's heat and moisture exchanger (HME) filter (3). [Color figure can be viewed at wileyonlinelibrary.com]

To assess the air pressure required for participants to produce tracheoesophageal speech and to determine whether increased muscle strength affects this pressure buildup, intratracheal air pressure was digitally measured during voice recordings using the FLUKE VT650 Gas Flow Analyzer (GFA). This GFA device measures air pressure per second in millimeters of mercury (mmHg). A study‐specific connector was designed and 3D‐printed by Oceanz (Ede, the Netherlands) using a Selective Laser Sintering (SLS) printer with polyamide 12 (Oceanz PA12 polished white; certified for medical application [ISO13485]). This connector enables the attachment of the GFA device to the participant's stoma adhesive (see Figure 2). This 3D‐printed connector consists of a ring that fits the ATOS stoma adhesive (1), a cone that accommodates the 1‐m plastic tube between the connector and the GFA device (2), and a front opening for the participant's HME filter (3). The participant first placed the 3D‐printed adapter onto the stoma adhesive and clicked their own HME filter into the adapter. The clinician attached the 1‐m plastic tube to the cone of the connector and connected the other end of the tube to the GFA device. Following this setup, the participant was given 5 min of rest to get used to the connector before the voice tasks were performed. For analysis, the tracheal pressure (in mmHg) was measured as the average value per participant over a 3‐s segment of a sustained vowel at neutral pitches. For consistency, this 3‐s segment was the same one utilized in the AVQI analysis. Additionally, the mean pressure was calculated for each entire task individually (including neutral, soft, low, high, and loud pitches).

#### 2.2.5 HRIM

To evaluate the pressure required in the (neo) pharynx and esophagus for producing tracheoesophageal speech, and to explore whether enhanced muscle strength impacts this pressure buildup, real‐time measurements in mmHg were taken during tracheoesophageal voicing using HRIM [32]. The Solar GI HRIM Solid State Trolley System (SN 18710476, Laborie, Enschede, the Netherlands) was used in combination with a 36‐sensor solid‐state catheter (Unisensor HRM K103659‐E‐118‐D, Laborie, Enschede, the Netherlands). The assessment was combined with videofluoroscopy (VFSS) and, therefore, performed at the radiology department.

**FIGURE 3:**
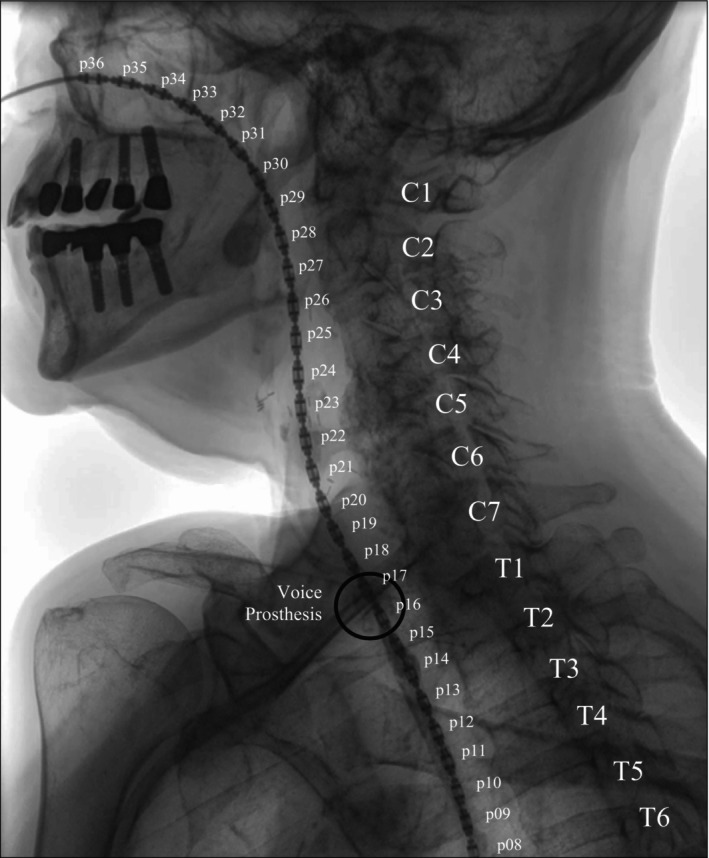
Videofluoroscopic image of a participant with the pharyngeal high‐resolution impedance manometry (HRIM)—catheter in situ.

Prior to the assessment, the participant, sitting in an upright position, was asked to select their preferred nostril for catheter insertion, which was then anesthetized with lidocaine spray. After approximately 5 min, the catheter was inserted 36 cm via the nose into the esophagus and secured with nasofix tape on the nose. The participant was then given an additional 5 min to get used to the catheter in their nose and pharynx. During this time, the radiology technician calibrated the CombiDiagnost R90 (Philips, Best, the Netherlands) and positioned the participant still sitting in an upright position in a lateral view see (Figure 3).

In the lateral view, the participant was instructed to swallow 10 cc of thin liquid contrast (Omnipaque 300 mg/mL combined with the Standardized Bolus Medium [SBMkit] product [Trisco, Pty. Ltd., Austrialia]) to coat the pharynx. Next, the participant was instructed to remain seated in an upright position and rotate their entire body approximately 20° to the right, creating a slightly angled view to improve residue detection in the (neo) pharynx, around the voice prosthesis, and in the esophagus. Following this, the participant was asked to sustain the vowel/a/on different extremes (neutral, soft, low, high, loud), each for 5 s. The raw pressure measurement data was exported to an ASCII (.asc) format and uploaded for analysis to the Swallow Gateway software (www.swallowgateway.com, version 2022; Flinders University, Adelaide, Australia). To prepare the analysis in Swallow Gateway, all voicing tasks were selected by drawing a region of interest (ROI) box in a single frame. For each task, 2 s of esophageal pressure data were extracted and analyzed. Since no pressures were measured in the (neo) pharynx during voicing, the ROI for each voicing task extended from the UES (at the C6 vertebral level) to approximately 12–13 cm distally within the esophagus. The mean pressure, measured in mmHg·cm·s, was then calculated for this ROI for each participant during each sustained vowel voicing task (neutral, soft, low, high and loud).

#### 2.2.6 Correlations Between Outcomes

To explore the relationships between the primary outcomes (AVQI, intensity, dynamic range, and PROMs) and secondary outcomes (tracheal and esophageal pressure), correlations were calculated.

#### 2.2.7 Statistical Analysis

The software program R (version 4.2.1) was used for all statistical analyses. Descriptive statistics were generated to summarize participant characteristics and results of primary outcomes (AVQI, Intensity, VHI‐10, V‐RQOL, and CPIB‐10). Continuous variables were summarized using the mean or median and range. To summarize the changes over time of continuous outcomes, linear mixed‐effects models (LME) were used, with time considered as a categorical variable. LME models were chosen for their ability to handle repeated measures, account for individual variability, and manage missing data points. The estimated marginal means from the model, with a corresponding 95% confidence interval (95% CI) were plotted along with the individual data.

Separate bivariate LME regressions were conducted for each outcome. Each model included a single outcome variable (*Y*), a predictor variable (*X*), and a random intercept for each participant. Prior to the analyses, all variables were standardized by subtracting the mean and dividing by the standard deviation, so the fixed‐effect beta of the model would reflect the correlation between the two variables. Correlations were displayed in a heatmap for comprehensive visualization. Correlations were interpreted as negligible (0.00–0.19), low (0.20–0.39), moderate (0.40–0.59), high (0.60–0.79), and very high (0.80–1.00). Because of the hypothesis‐generating nature of this analysis, we did not consider statistical significance levels.

## 3 Results

All 20 participants completed the T0 and T1 measurements. Nineteen participants completed the T2 measurements because one participant (S12) declined due to medical reasons unrelated to the study.

### 3.1 Voice Quality

None of the outcomes for voice quality (AVQI, dynamic range and intensity) showed significant changes over time in the estimated marginal means; see Table 2 (and Appendices S2–S4). However, on the individual participant level, some clinically relevant changes in the AVQI were noted (three better and three worse) (see Table 3).

**TABLE 2:** LME model values of voice parameters.

Voice parameters	T0, PMM (95% CI)	T1, PMM (95% CI)	T2, PMM (95% CI)
Voice quality			
AVQI	8.25 (7.84, 8.67)	8.02 (7.60, 8.44)	8.25 (7.82, 8.67)
Intensity measured in dB			
Sustained vowel/a/:			
Neutral	66.63 (63.63, 69.63)	67.82 (64.82, 70.82)	67.81 (64.79, 70.83)
Soft	57.60 (54.91, 60.30)	57.47 (54.83, 60.12)	58.59 (55.89, 61.28)
Low	63.00 (59.95, 66.04)	64.10 (61.00, 67.19)	62.38 (59.23, 65.53)
High	65.93 (62.94, 68.91)	68.40 (65.41, 71.39)	67.93 (64.91, 70.95)
Loud	75.69 (72.69, 77.71)	75.43 (72.92, 77.95)	76.38 (73.84, 78.91)
SVintmean	68.69 (65.88, 71.50)	70.10 (67.29, 72.91)	69.05 (66.20, 71.89)
Dynamic range	17.74 (14.90, 20.58)	17.96 (15.18, 20.74)	17.84 (15.01, 20.68)
Clinician‐rated perceptual outcomes measured with a five‐points scale			
Tonicity	2.98 (2.50, 3.47)	3.00 (2.51, 3.49)	3.07 (2.58, 3.56)
Intelligibility	3.05 (2.68, 3.42)	2.88 (2.51, 3.25)	3.00 (2.63, 3.37)
PROMs			
VHI‐10	22.30 (18.84, 25.76)	21.15 (17.69, 24.61)	19.29 (15.81, 22.77)
V‐RQOL	60.62 (51.87, 69.38)	64.50 (55.75, 73.25)	70.95 (62.15, 79.75)
Social–emotional	75.94 (65.55, 86.32)	78.44 (68.05, 88.82)	82.61 (72.16, 93.06)
Functional	70.25 (64.93, 75.57)	73.12 (67.81, 78.44)	76.73 (71.38, 82.08)
CPIB‐10	16.60 (13.58, 19.62)	18.05 (15.03, 21.07)	18.78 (15.73, 21.82)
Tracheal pressure measured with the FLUKE in mmHg			
Sustained vowel/a/:			
Neutral	26.93 (22.47, 31.39)	28.13 (23.67, 32.60)	24.70 (20.18, 29.22)
Soft	16.29 (12.90, 19.68)	14.71 (11.39, 18.03)	16.66 (13.27, 20.04)
Low	27.20 (22.62, 31.78)	22.23 (17.58, 26.87)	22.68 (17.96, 27.39)
High	26.88 (22.07, 31.70)	26.97 (22.16, 31.79)	28.48 (23.59, 33.37)
Loud	51.67 (43.39, 59.95)	49.85 (41.57, 58.12)	49.38 (40.98, 57.78)
SVpmean	27.23 (22.30, 32.17)	27.98 (23.05, 32.92)	23.97 (18.96. 28.98)
Continued speech	21.00 (16.83, 25.16)	20.55 (16.38, 24.71)	21.44 (17.22, 25.66)
Esophageal pressure measured with HRIM in mmHg cm s			
Sustained vowel/a/:			
Neutral	361.13 (247.03, 475.24)	384.69 (273.02, 496.35)	376.47 (262.37, 490.57)
Soft	268.43 (193.71, 343.16)	207.88 (133.15, 282.60)	193.69 (120.69, 266.68)
Low	299.90 (215.56, 384.240	271.85 (187.57, 356.14)	297.36 (213.07, 381.64)
High	421.59 (309.86, 533.33)	378.00 (268.86, 487.14)	414.02 (305.07, 522.97)
Loud	496.26 (370.04, 622.49)	546.45 (426.90, 666.01)	541.76 (415.72, 667.80)

Abbreviations: PMM, Predicted Marginal Mean; AVQI, Acoustic Voice Quality Index; CPIB‐10, Communication and Participation Item Bank; HRIM, high‐resolution impedance manometry; PROM, patient‐reported outcome measurement; SLP, speech‐language pathologist; SVintmean, mean intensity of 3‐s sustained vowel neutral; SVpmean, mean pressure of 3‐s sustained vowel neutral; T0, baseline; T1, short‐term results after 6 weeks of training with the SEA2.0; T2, long‐term results after 8 weeks of rest; VHI‐10, Voice Handicap Index; V‐RQOL‐10, Voice‐Related Quality of Life.

**TABLE 3:** Summary of primary outcomes at participant level: increases, decreases, and clinical relevance across time points (T2 vs. T0). [Color table can be viewed at wileyonlinelibrary.com]

Summary of differences on primary outcomes at participant level: T2 vs. T0																				Number of clinically relevant changes (%)		Mean difference of all participants (SD)	
Participants	S01	S02^a^	S03^a^	S04^a^	S05	S06	S07	S08	S09^a^	S10	S11	S14^a^	S15	S16	S17	S18	S19^a^	S20	S21	↓	↑	↓	↑
Primary outcomes																							
AVQI	↓	=	↑	↓	↓	↑	=	↑	↓	=	=	↑	=	↓	↓	=	=	↑	↓	3 (15)	3 (15)	−0.7 (0.6)	1.1 (0.9)
Dynamic range (dB)	↑	↑	↑	↓	↓	↓	↓	↓	↓	↑	↑	↑	↓	↑	↓	↑	↑	↑	↓	NA	NA	−5.5 (4.1)	5.1 (3.7)
SVintmean (dB)	↑	↓	↓	↑	↓	↓	↑	↓	↑	↓	↑	↑	↑	↑	↑	↓	↑	↑	↓	NA	NA	−3.5 (2.1)	4.6 (3.2)
VHI‐10	↓	↓	↑	↓	↓	↓	=	↓	↓	↓	↓	↓	↓	↓	↑	↓	=	↓	↓	7 (35)	1 (5)	−4.1 (2.3)	4.0 (2.0)
V‐RQOL	↑	↑	↑	↓	↑	↑	=	↑	=	↑	↑	↑	↓	↑	↑	↑	↑	↑	↑	0 (0)	6 (30)	−8.8 (5.3)	14.3 (8.2)
CPIB‐10	↓	↑	↑	↑	↑	↑	↑	↑	=	↓	↓	↑	↑	↑	↓	↑	↑	↑	↓	NA	NA	−3.6 (2.8)	4.5 (4.4)

*Note*: Legend of colors: Dark green, clinically relevant improvement; Light green, improved but not clinically relevant; Dark red, clinically relevant worsened; Orange, worsened but not clinically relevant. Clinically relevant differences: 5 points on VHI‐10 [26], 15 points on V‐RQOL [29], and 0.95 points on AVQI [25]. For CPIB‐10, Dynamic Range and Intensity vowel/a/neutral, no clinically relevant values are available.

Abbreviations: AVQI, Acoustic Voice Quality Index; Dynamic range, mean intensity in dB of loud vowel/a/minus soft vowel/a/; SVintmean, mean intensity in dB of 3‐s sustained vowel on neutral pitch; =, equal; ↑, increase; ↓, decrease.

^a^ Participants with a history of stenosis and dilatations.

### 3.2 Clinician‐Rated Perceptual Voice Tonicity and Intelligibility

Table 2 shows the clinician‐rated perceptual scores for voice tonicity and intelligibility. One SLP, having less than 5 years of experience, was considered an outlier and had to be excluded from the tonicity part of the experiment due to inconsistent and scattered results. Another SLP had technical issues during the assessment, resulting in the omission of all responses to the voice tonicity question, leaving the assessments of three SLPs for inclusion in the tonicity analysis. No significant changes were observed in the clinician‐rated perceptual tonicity of the tracheoesophageal voice; see Appendix S5.

The clinician‐rated perceptual intelligibility scores were completed by all five SLPs, with heterogeneous outcomes among them; see Appendix S6. However, no significant changes were observed in the clinician‐rated perceptual intelligibility of tracheoesophageal speech.

### 3.3 PROMs

None of the outcomes for PROMS showed significant changes over time in the estimated marginal means; see Table 2 (and Appendices S7–S9). However, on the individual patient level, some clinically relevant changes were noted for one or two of the outcomes in 11 patients (see Table 3).

### 3.4 Tracheal Manometry

**FIGURE 4:**
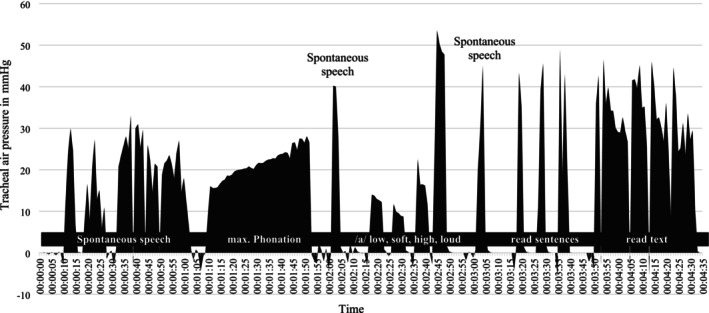
Example of tracheal (air) pressure (in mmHg) per second for various tracheoesophageal voice tasks, measured using the FLUKE VT590 gas flow analyzer. During inhalations, the pressure drops below zero.

Figure 4 illustrates the tracheal air pressure of one participant during various tracheoesophageal tasks, showing a drop below zero during inhalation and an increase in pressure before and during the tasks. Table 2 (and Appendices S10–S14) shows the tracheal pressure (in mmHg) during sustained vowel on neutral, soft, low, high, and loud pitch and during continued speech. The measured tracheal air pressure during a neutral sustained vowel was around 67 mmHg, approximately 58 mmHg for soft, 63 mmHg for low, 66 mmHg for high, and 76 mmHg for loud. None of the outcomes for tracheal manometry showed significant changes over time in the estimated marginal means; see Table 2. However, on the individual patient level, some changes were noted (in both directions); see Appendix S10.

### 3.5 HRIM

A total of 17 out of 20 participants completed the HRIM assessment. One participant was excluded from the analysis due to a gastric pull‐up (S21), which prevented proper marker placement. The third participant (S19) declined the HRIM due to fear of the catheter and only underwent the VFSS.

**FIGURE 5:**
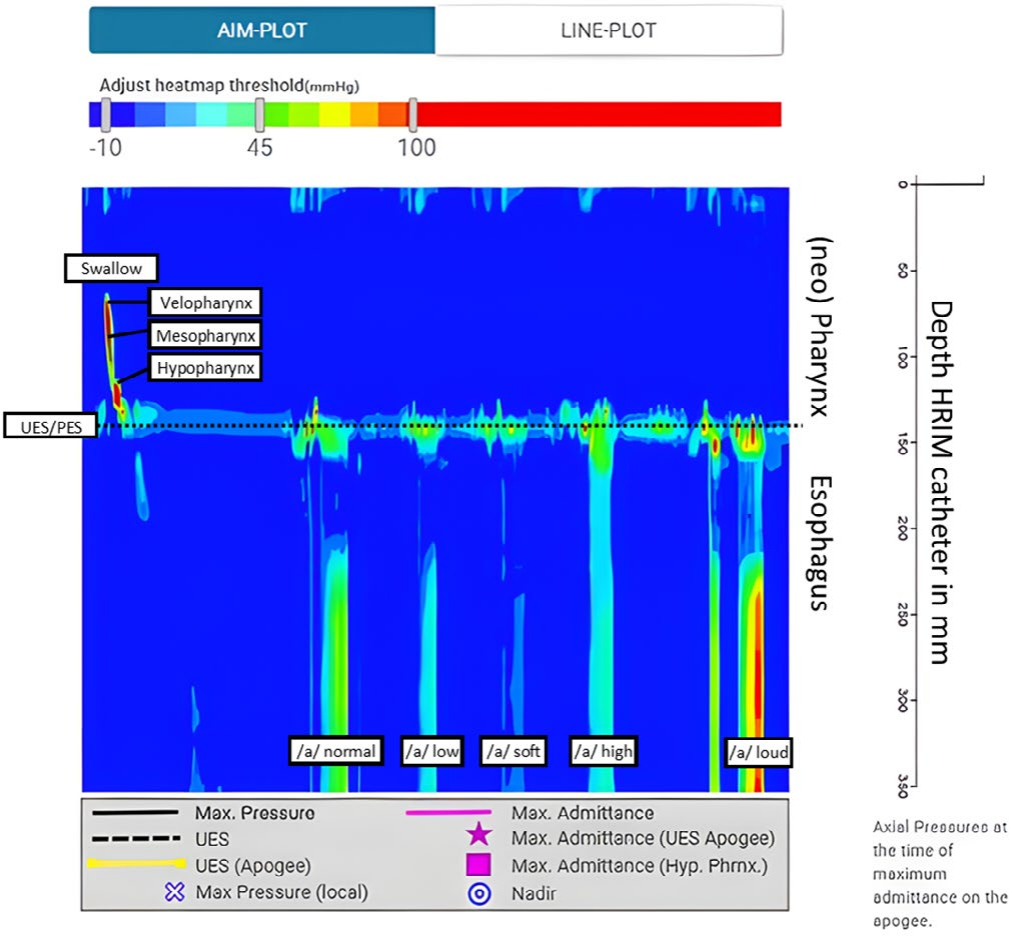
Color plot of HRIM measurement during swallow and tracheoesophageal voicing. Blue, no pressure (~0 mmHg); green, low pressure (~45 mmHg); red, high pressure (~100 mmHg). [Color figure can be viewed at wileyonlinelibrary.com]

Figure 3 displays a video fluoroscopic image of a participant with the HRIM catheter in situ, and in Figure 5, a color plot of the HRIM measurement is displaying both a swallow and tracheoesophageal voicing. It is highlighted in this color plot that no pressure is generated in the (neo) pharynx during voicing. The plot illustrates the variations in esophageal pressure (below the UES) during sustained vowels/a/at neutral, soft, low, high, and loud pitches in tracheoesophageal voicing, clearly demonstrating that each task requires different pressures in the esophagus (see Figure 5). The measured esophageal pressure during a neutral sustained vowel in this sample was around 361 mmHg, approximately 268 mmHg for soft, 300 mmHg for low, 422 mmHg for high, and 496 mmHg for loud.

Table 2 (and Appendices S10, S15–S19) shows the outcomes of the LME models on esophageal pressures, measured with HRIM during tracheoesophageal speech over time. As shown in Appendices S15–S19, some participants were unable to sustain a vowel sound during the assessment, resulting in variations in the number of participants available for each task and measurement due to the catheter's presence in the (neo) pharynx and esophagus. No significant changes have been found in all pressures within the esophagus during tracheoesophageal speech between T0, T1, and T2. However, during the neutral and loud sustained vowels, esophageal pressures appeared to increase slightly after training, while pressures for soft, low, and high vowels decreased (see Table 2).

### 3.6 Correlations Between Outcomes

**FIGURE 6:**
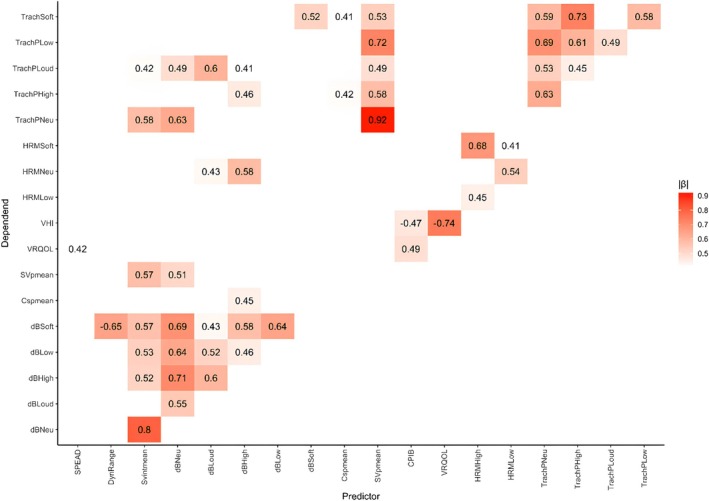
Heat map for *β* estimates from standardized linear mixed effect regression models for all parameter pairs. Formula: Dependent − Predictor + (1|Subject). The numbers are the coefficient estimates (*β*) for all pairs where the absolute value of I was ≥ 0.4. CPIB, Communication Participation Item Bank; CSpmean, mean tracheal pressure of 3 s of continued speech; dBSoft/Low/High/Loud/Neu, intensity in dB of sustained vowel/a/on soft/low/high/loud/neutral pitch; DynRange, dynamic range (maximal‐minimal) in intensity; HRMSoft/Neu/Low/High/Loud, esophageal pressure measured with high‐resolution (impedance) manometry during sustained vowel/a/on soft/neutral/low/high/loud pitch; SVpmean, mean tracheal pressure of 3 s of the sustained vowel/a/on neutral pitch; TrachPSoft/Low/Loud/High/Neu, Tracheal air pressure measured in mmHg with the FLUKE during sustained vowel/a/on soft/low/loud/high/neutral pitch; VHI, Voice Handicap Index‐10; VRQOL, Voice‐Related Quality of Life. [Color figure can be viewed at wileyonlinelibrary.com]

Figure 6 displays the heat map of the LME regression models for all primary and secondary parameters, highlighting correlations with an R value above 0.4. Notable correlations were observed between intensity and pressure measurements, particularly within the high (0.60–0.79) range, such as between various dB measures (e.g., dBSoft, dBNeutral) and pressure values (e.g., Tracheal Pressure Neutral/a/[TrachPNeu]). Stronger correlations (0.80–0.89) were found between sustained vowel mean intensity (SVintmean) and dBNeutral, and between the PROMS VHI‐10 and VRQOL. The strongest correlation, exceeding 0.9, was found between two variables measuring tracheal pressure (SVpmean and TrachPNeu).

## 4 Discussion

This exploratory study aimed to investigate tracheoesophageal voicing following a resistance‐based dysphagia rehabilitation program, using a multidimensional evaluation approach. Assessment of the various primary outcome measures, that is, AVQI, intensity in dB, clinician‐rated voice tonicity and intelligibility, and PROMS indicates that tracheoesophageal voice quality on average is not significantly changed following this muscle training for dysphagia. However, at the participant level, there were several interesting clinically relevant changes, which warrant discussion.

Voice quality was assessed using the acoustic analysis AVQI. In our participants, the mean AVQI score was 8.25 (the maximum [worst] being 10). The cut‐off score of the AVQI is, for laryngeal voice, above 2.95; however, it shows that all laryngectomized participants' voices must be classified as highly distorted. Voices were more distorted than in a previous study from our Institute, in which the mean AVQI score for laryngectomized participants 12 months post‐laryngectomy was 5.99 (SD 2.94) [10]. The higher AVQI scores in our study most likely can be explained by selection bias. Because the focus of the Clinical Phase II trial was resistance‐based swallowing rehabilitation, participants were only included because of their self‐reported swallowing problems. However, 45% of the included participants also reported voicing problems at T0. Since after total laryngectomy the functions swallowing and voicing share the neopharynx/neoglottis as passageway and sound generator, respectively, this relatively high AVQI is not surprising. It is likely that self‐selection of participants who signed up for this study experiences more swallowing and voice complaints than the general TL population. The observation that three patients showed a clinically relevant improvement of more than 0.95 after trainingshould, however, be interpreted with caution, as the AVQI has not yet been validated for post‐laryngectomy voicing. Additionally, this improvement is not consistently reflected in the VHI‐10 scores, as participants with improved AVQI did not necessarily show improvements in their VHI‐10 results, and three patients with a worsened AVQI showed several improvements in the PROMs, as discussed below.

The intensity of the sustained vowel, measured in dB, remained stable over time. The mean measured intensity was averaging around 69 dB, which is comparable to literature [33, 34, 35]. However, compared to another study from our institute, our average was higher [36]. That study reported the outcomes of expiratory muscle strength training (EMST) in laryngectomized individuals [36]. The mean vocal intensity at baseline of 26.4 dB (SD 5.6) increased to 31.9 dB (SD 3.7) after 4 weeks of EMST training [36]. This suggests that the increase in intensity might be more influenced by respiratory muscle training than by factors related to swallowing muscle training.

Tonicity and intelligibility ratings by the SLPs showed that the participants group mean scores remained stable around *normotonic* and *neither poorly nor highly intelligible* levels. However, the heterogeneity in individual SLP ratings and the fact that there was one outlier makes drawing clear conclusions at the participant level challenging. Another option for clinician‐rated outcomes that could have been used is the Sunderland Tracheoesophageal Perceptual Scale (SToPS), which assesses tracheoesophageal voice quality; however, it was not applied in our research because of the need for specialized training, its time‐consuming nature with many components, and concerns about the reliability of results with a limited number of trained raters [16, 37, 38]. This calls for future automated tools for tonicity and intelligibility assessment to improve the consistency and accuracy of tonicity and intelligibility assessment, possibly using artificial intelligence [39].

With respect to the PROMs outcomes, compared to earlier studies, our participants had worse VHI‐10 [36, 40] and V‐RQOL [41, 42] scores, which align with the poorer AVQI outcomes observed in our study. For instance, the mean VHI‐10 score reported by van Sluis et al. was 15.8 in laryngectomized patients 12 months postsurgery, whereas our population had a worse mean score of 22.3. In addition, the mean VRQOL in laryngectomized patients reported by Moukarbel et al. was 76.5 versus a worse mean score of 60.62 in our population at T0 and 70.95 at T2 [43]. This suggests that both the objective and subjective measures of voice quality in our participants were worse than those reported in other studies. Therefore, again, the results of this study should be interpreted with caution, as they obviously have been influenced by the (swallowing) selection bias.

It is noteworthy, though, that in total, 14 participants showed clinically relevant improvements on the AVQI or (one of the) PROMs. A clinically relevant improvement is defined as a better scores on objective AVQI and/or fewer voice complaints on the subjective PROMS. This improvement may be a neuroplasticity phenomenon, suggesting that the increased muscle strength and improved swallowing, as shown in the Clinical Phase II trial, also benefit patient‐reported voicing outcomes in some of the patients [8]. However, because the surgery certainly has caused some denervation of the musculature in the pharyngeal region, in some patients the occurrence of neuroplasticity is unlikely any longer. Part of the voicing improvements at the participant level may also result from their swallowing improvements; participants' likely experienced greater well‐being, which may reflect in an optimistic mindset when reporting their voice‐related QoL in the VHI‐10, V‐RQOL as well as increased participation in daily activities (CPIB‐10). The correlation analysis highlights that the VHI‐10 and V‐RQOL are highly correlated with each other and moderately correlated with the CPIB‐10 but show no correlation with the measured physical outcomes. This suggests that participants may perceive improvements in their condition and experience benefits of their rehabilitation, leading to better scores on PROMs.

To better understand and/or explain the primary findings, tracheal pressures during tracheoesophageal speech were investigated. Although the results for tracheal pressures did not show significant changes over time, there appeared to be a slight decrease in mean tracheal pressures during sustained vowels and continued speech. In an earlier study conducted at our institute, tracheal air pressures (in mmHg) were measured during sustained vowels and while playing a brass instrument [34]. The air pressures measured in the current study are higher than those reported in that study, possibly due to the good tracheoesophageal voice and swallowing of that patient and a different method of measuring equipment. We used the FLUKE GFA with a specific 3D‐printed adapter to connect the adhesive to the HME and FLUKE GFA via a 1 m flexible plastic tube, rather than a digital manometer that was tightly attached to a peristomal adhesive. Currently, there is limited reference data on tracheal air pressure in relation to voice quality, and the interpretation of these pressures remains uncertain. In the literature, we found that Evangelista et al. measured intratracheal pressures in cmH_2_O in total laryngectomy patients and found that higher pressure was linked to shorter phonation times and poorer voice quality [44]. The pressures reported in that paper, when converted from cmH_2_O to mmHg, are much lower than those found in our study. Furthermore, the measurement methods used were quite different, making direct comparison impossible. Additionally, drawing meaningful conclusions about whether higher or lower tracheal pressures during certain voicing tasks reflect an improvement or decline in vocal quality remains challenging. Since the correlation analysis suggests that tracheal air pressure correlates with voice intensity, we might hypothesize that if participants can produce higher intensity (dB) voices with less pressure, a reduction in pressure could indicate an improvement in vocal efficiency. This hypothesis offers a promising direction for future research into understanding and optimizing vocal production using the FLUKE GFA.

To understand what is occurring in the (neo) pharynx and esophagus, HRIM was assessed. The HRIM results revealed distinct differences in the pressures generated in the esophagus during various tasks for tracheoesophageal voicing. It appeared that pressure builds up below the voice prosthesis, while the vibrating PE segment, located above the voice prosthesis, did not show any measurable pressure on the pressure plots. The pressure buildup in the esophagus below the voice prosthesis might be a result of the coordination and technique patients must use to speak, directing air into the (neo) pharynx instead of the stomach. Consequently, it is reasonable to assume that they need to generate more pressure during higher and louder speech compared to normal, soft, or low‐level speech. However, results showed a slight, though not significant, decrease in the esophageal pressure buildup during the low, soft, and high sustained vowels/a/. This may be attributed to improved control and coordination of the muscles after following the resistance‐based swallowing therapy [8]. Conversely, for the sustained vowel/a/in neutral and loud conditions, participants seem to build up more pressure in the esophagus, although this increase also was not significant. From the correlation calculation, the esophageal pressure showed correlations between the different tasks but not with other outcomes, due to its more task‐dependent nature and the fact that it reflects processes unrelated to vocal function.

Our HRIM results showed higher pressures than those reported in the literature, which may be attributed to the differing measurement methods used; we employed HRIM, while the other studies utilized perfusion manometry [33, 45]. The study by Takeshita‐Monaretti et al. indicated that the middle and distal regions of the esophagus demonstrated compliance, which facilitated adjustments in intensity, and revealed no significant correlation between vocal intensity and esophageal pressure [33]. In contrast, Reis et al. reported a positive correlation between pressure amplitude in the proximal and distal esophagus and increased dynamic extension [45]. They found that individuals with normal dynamic extension exhibited greater contraction amplitude in the proximal esophagus compared to those with dynamic extension below the expected values for their age [45]. In light of these findings, our study found only moderate correlations between loud and high vocal intensity and esophageal pressure measured during sustained vowel on neutral pitch, meaning that the correlations between these two aspects remain unclear.

This study had several limitations. First, this study focused on a small group of patients with self‐reported dysphagia, which limits the generalizability of the findings to broader patient populations. Additionally, this group was even more impaired than average, as indicated by worse scores on PROMs. Second, the outcome measures used have not been validated for laryngectomized individuals, and it is unknown whether the values for clinical relevance are applicable to this specific patient population. Additionally, the measurements of tracheal and neopharyngeal pressures are new in this research area, and reference values are lacking. Another limitation of the tracheal pressure measurement performed with the FLUKE is its one‐pressure‐per‐second rate, which may have resulted in the loss of potentially valuable information.

## 5 Conclusion

In conclusion, in this specific sample of laryngectomized patients with self‐reported dysphagia, following a 6‐week resistance‐based rehabilitation program, no significant changes were found in objective and subjective voice quality and intelligibility, nor in tracheal and neopharyngeal pressures during voicing. Although at the participant level, there were several clinically relevant changes, overall, this indicates that tracheoesophageal speech on average is not affected by the resistance‐based swallowing therapy.

## Ethics Statement

The current study was part of the approved Clinical Phase II trial (METC21.0904/N21STL). The guidelines of the Helsinki Declarations were followed.

## Consent

Written informed consent was obtained from each participant before participation.

## Conflicts of Interest

The authors declare no conflicts of interest.

## Supporting information


**Data S1.**Supporting Information.

## Data Availability

The data set generated and analyzed during the current study are available from the corresponding author upon reasonable request.
