# Supplementary material for: Tracheoesophageal Voicing Following Resistance‐Based Dysphagia Rehabilitation: An Exploratory Multidimensional Assessment
Source: Head Neck. 2025 Mar 25;47(8):2209–22. doi: 10.1002/hed.28136 (PMC12248270; doi:10.1002/hed.28136)

# Appendix

## Appendix 1: Perceptual evaluation form


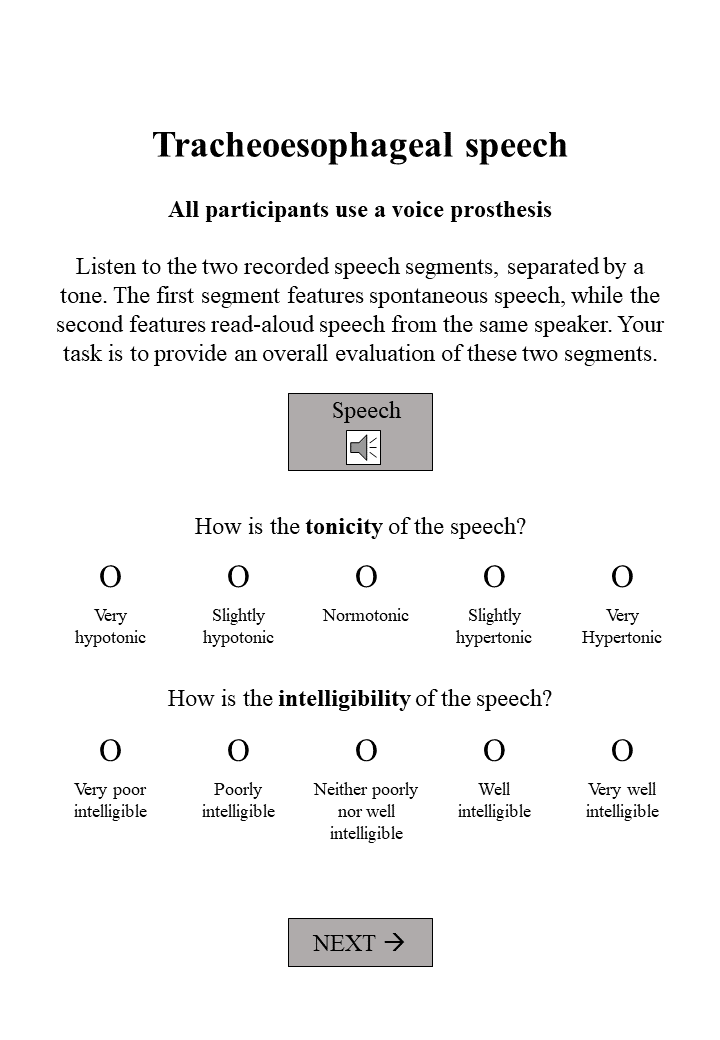


Abbreviation: this perceptual evaluation form has been translated from Dutch into English for this paper.

## Appendix 2: LME model of the AVQI values

**
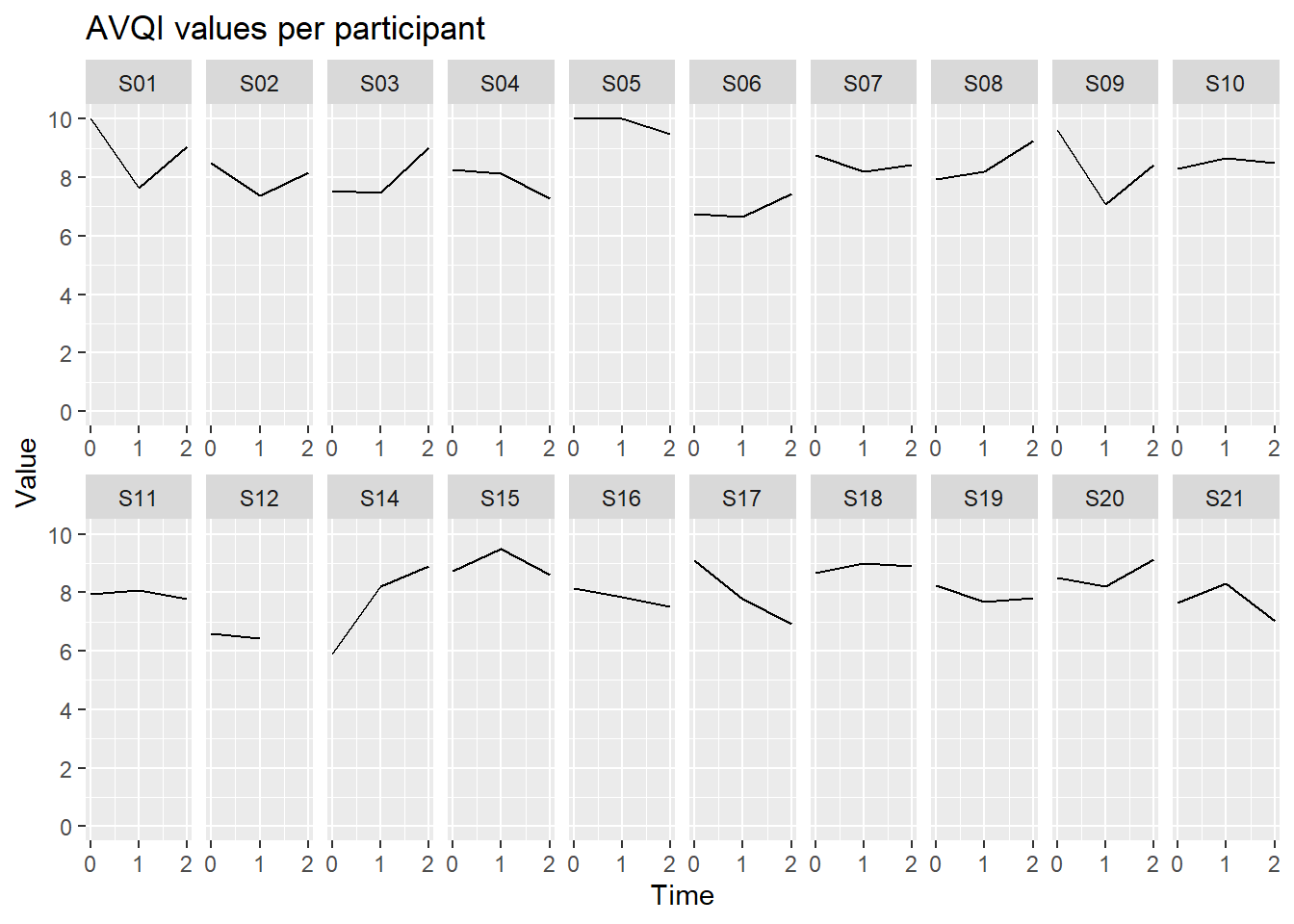
**

**
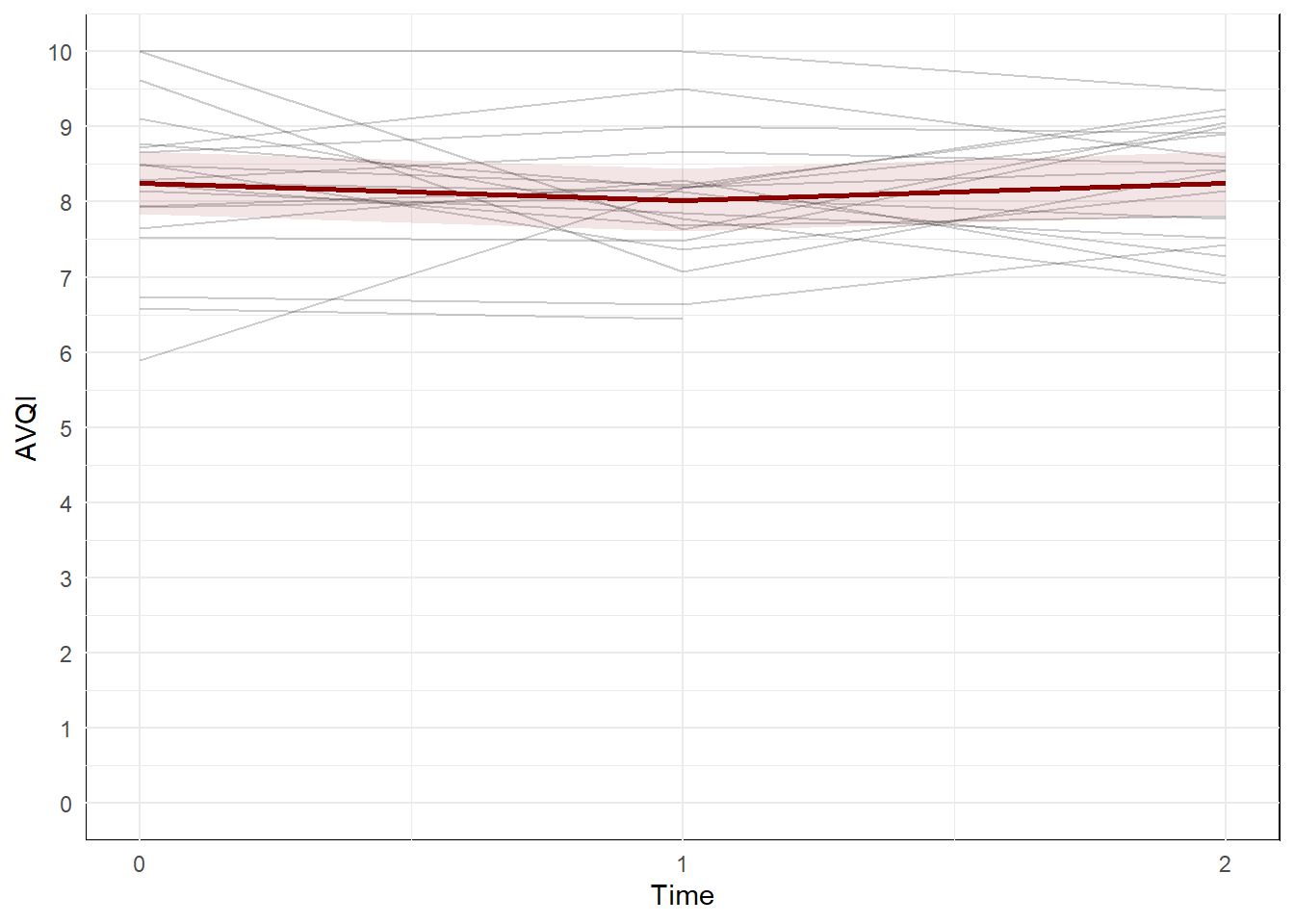
**

**
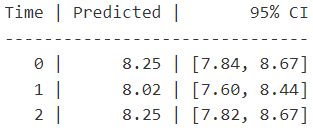
**

## Appendix 3: LME model on maximum intensity (dB) of sustained vowel /a/

**
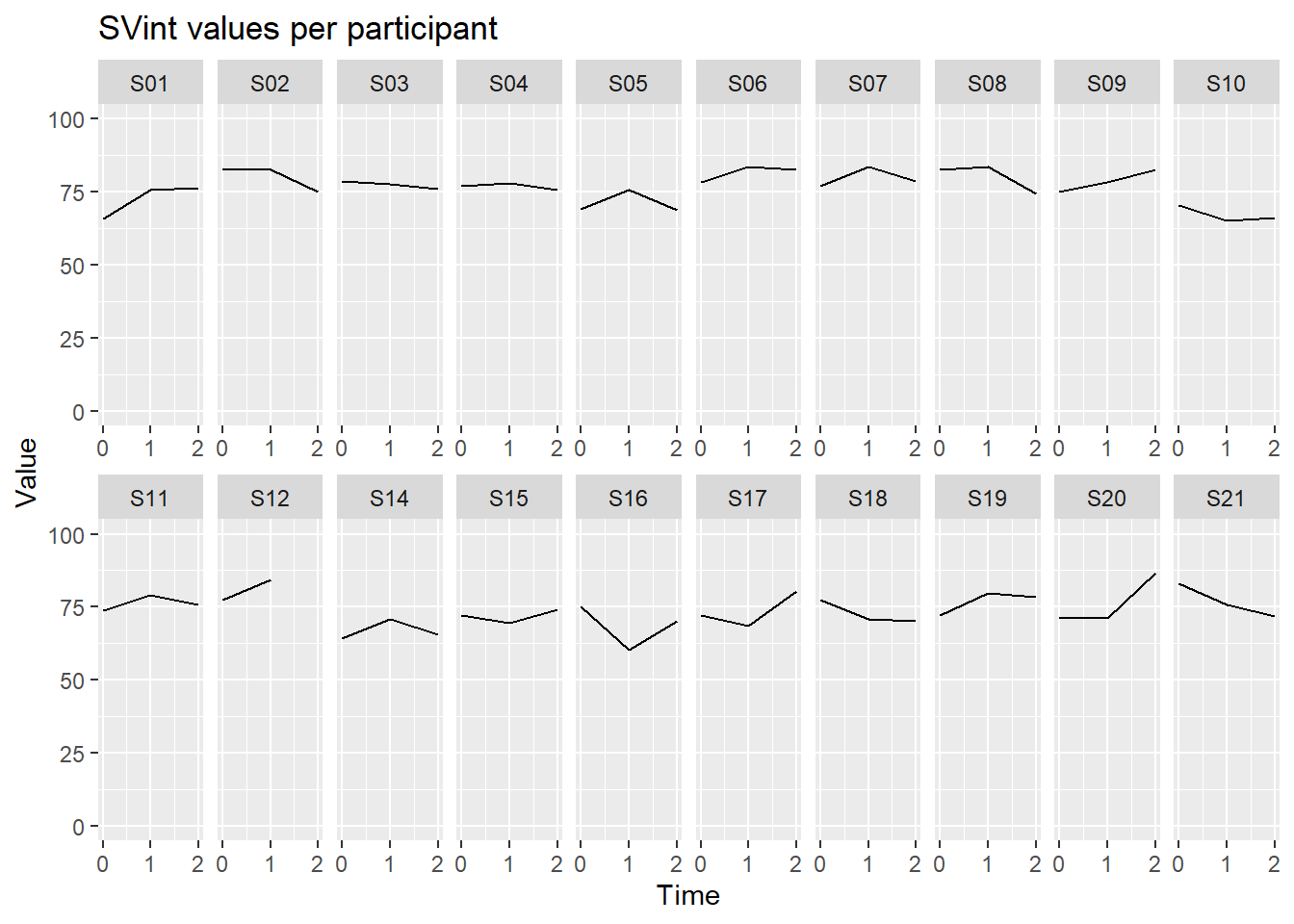
**

**
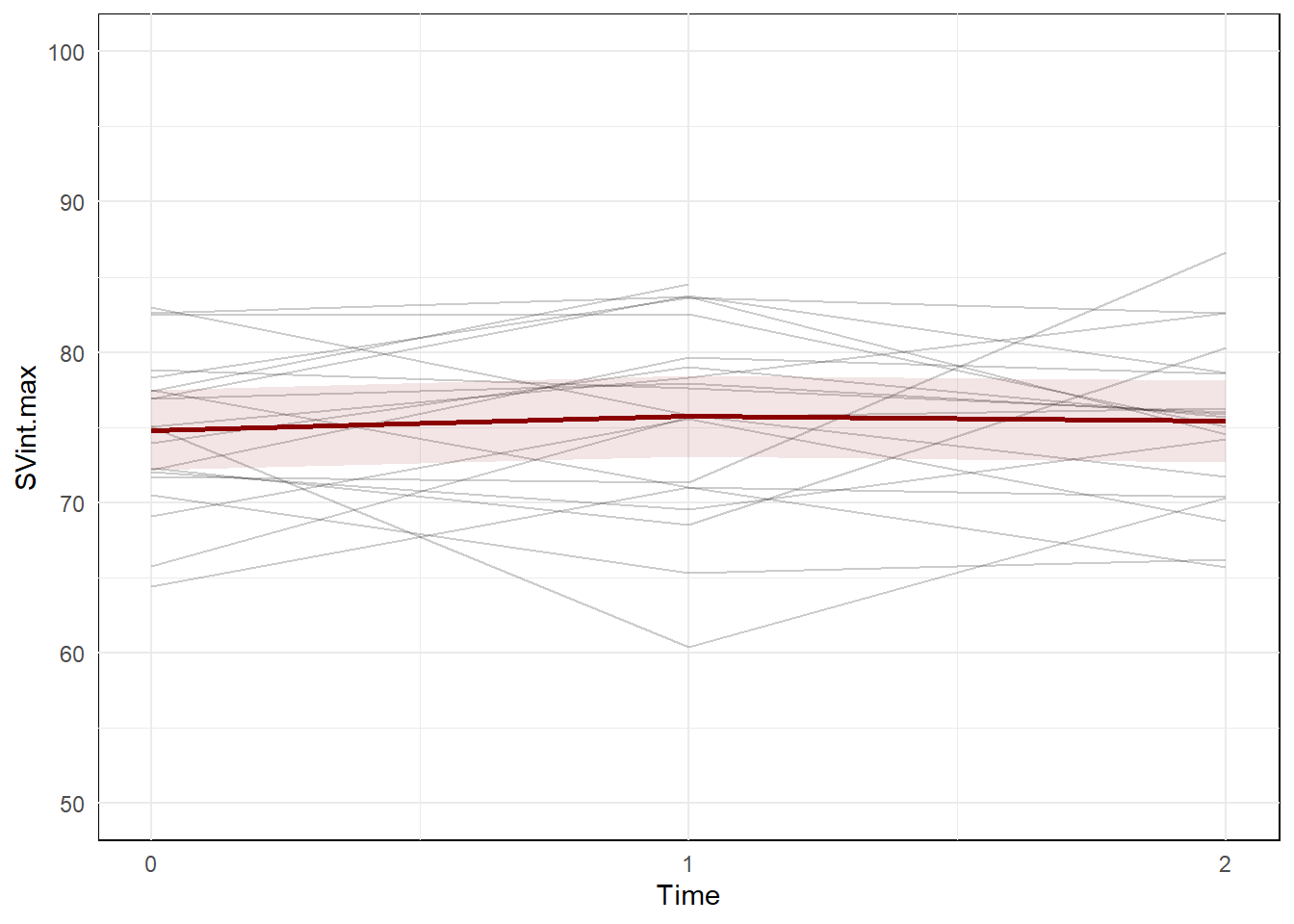
**

**
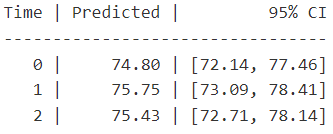
**

## Appendix 4: LME model on mean intensity (dB) of sustained vowel /a/

**
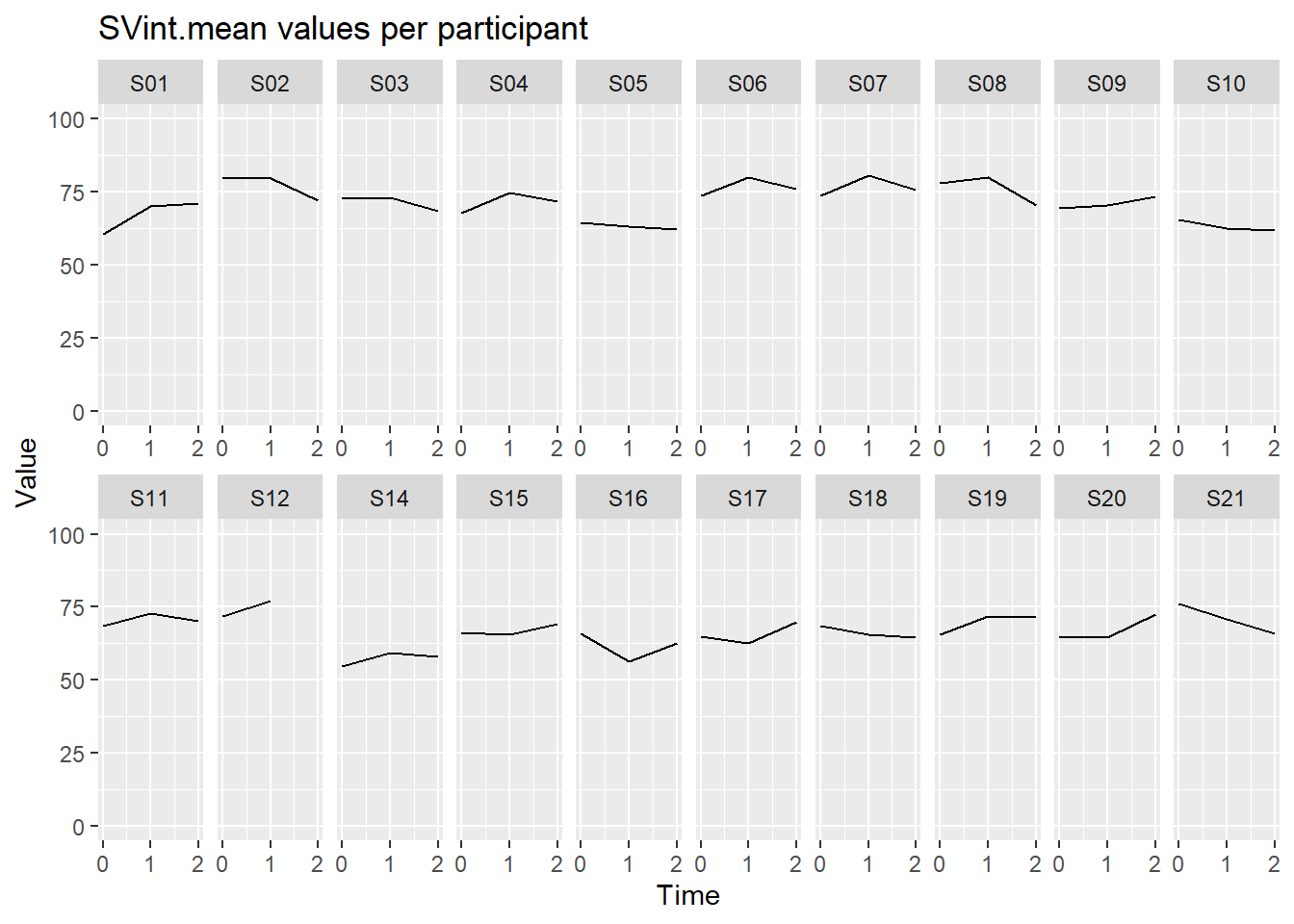
**

**
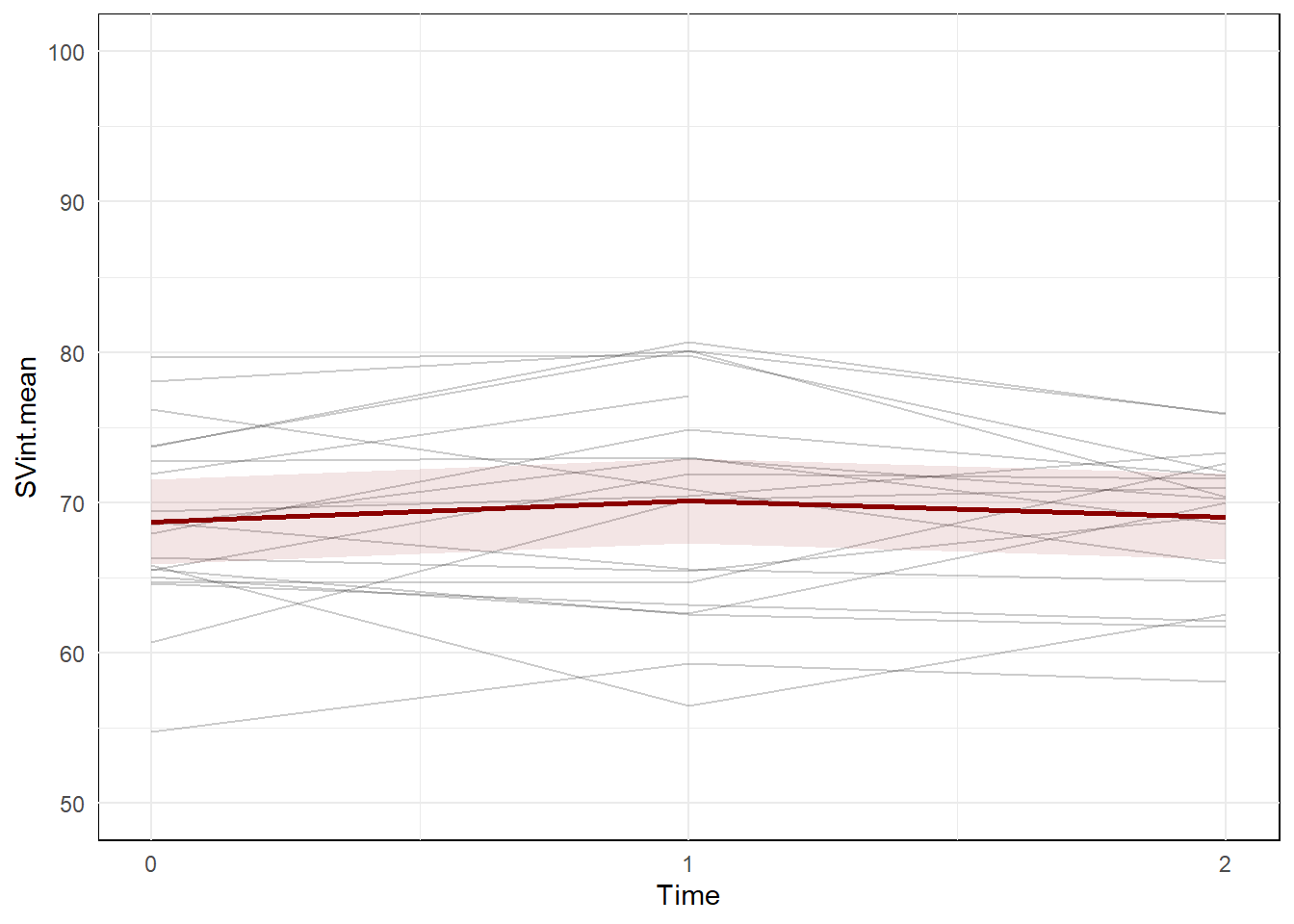
**

**
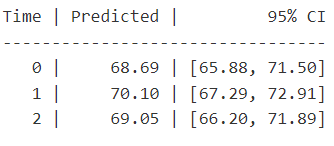
**

## Appendix 5: LME model on Likert Tonicity scores


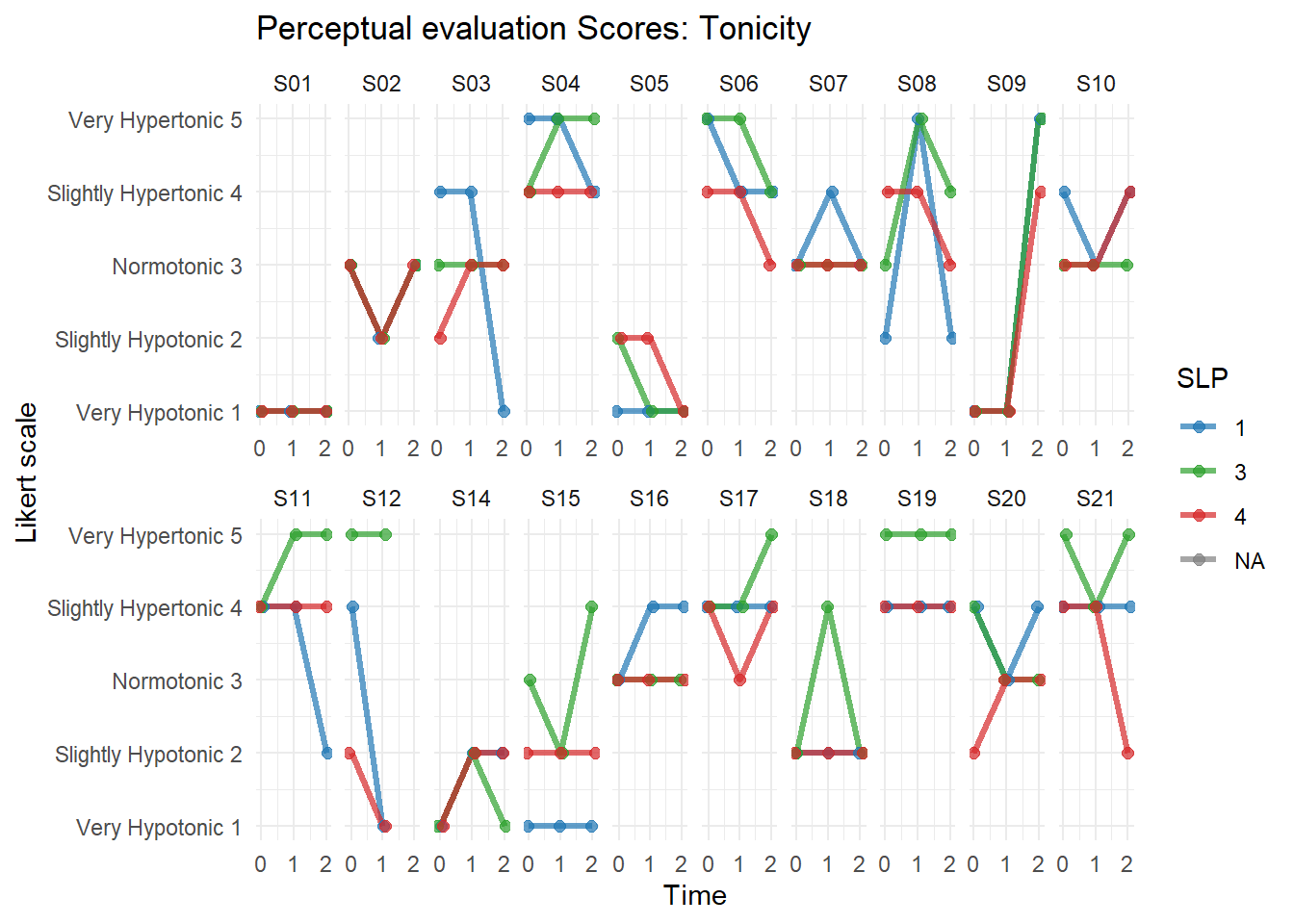


**
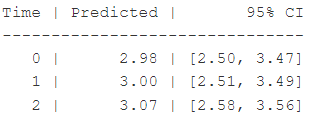
**

## Appendix 6: LME Model of the Likert Intelligibility scores


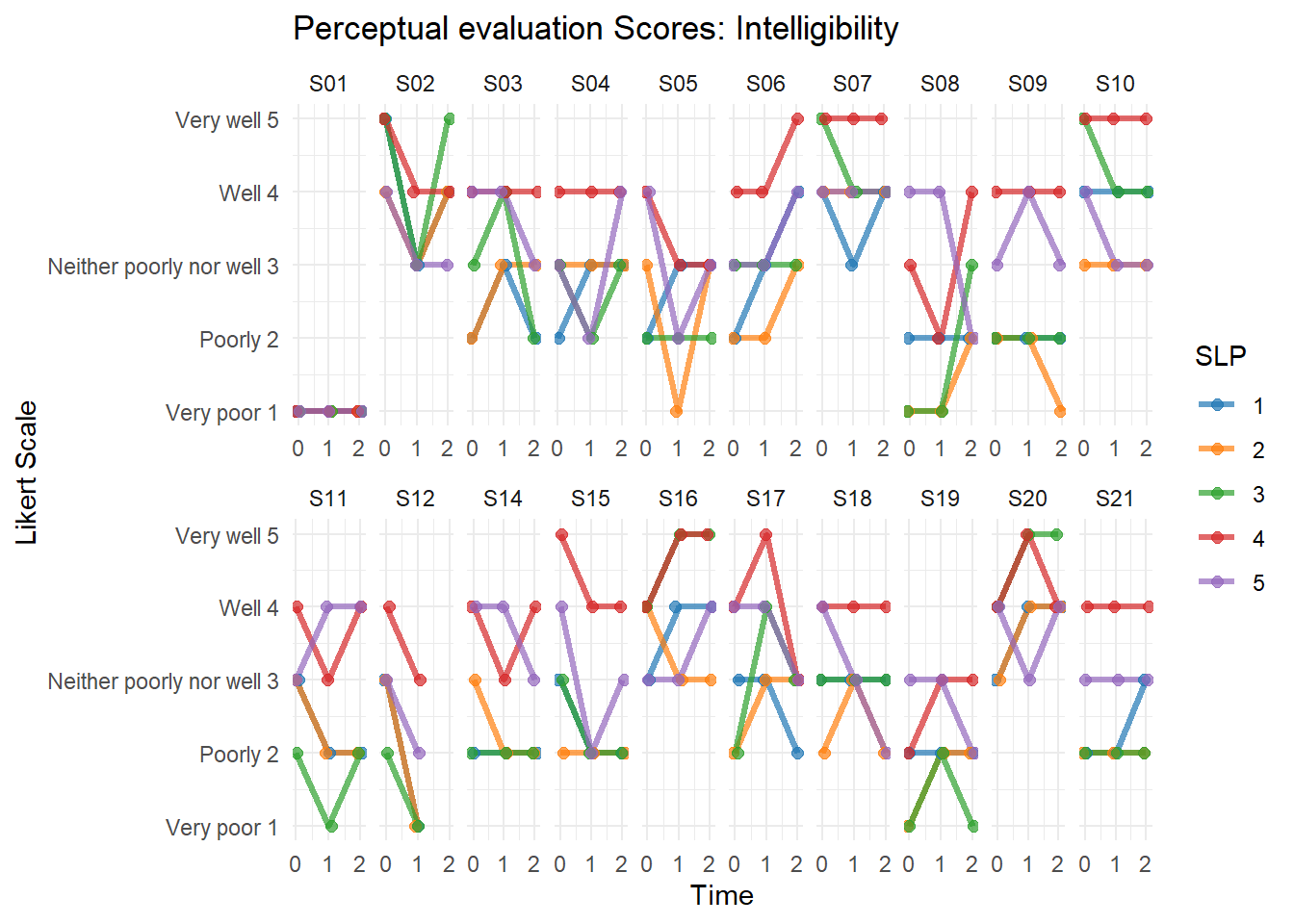


**
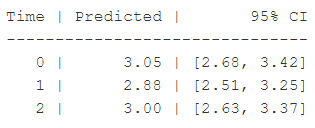
**

## Appendix 7: LME Model of the PROM: VHI-10


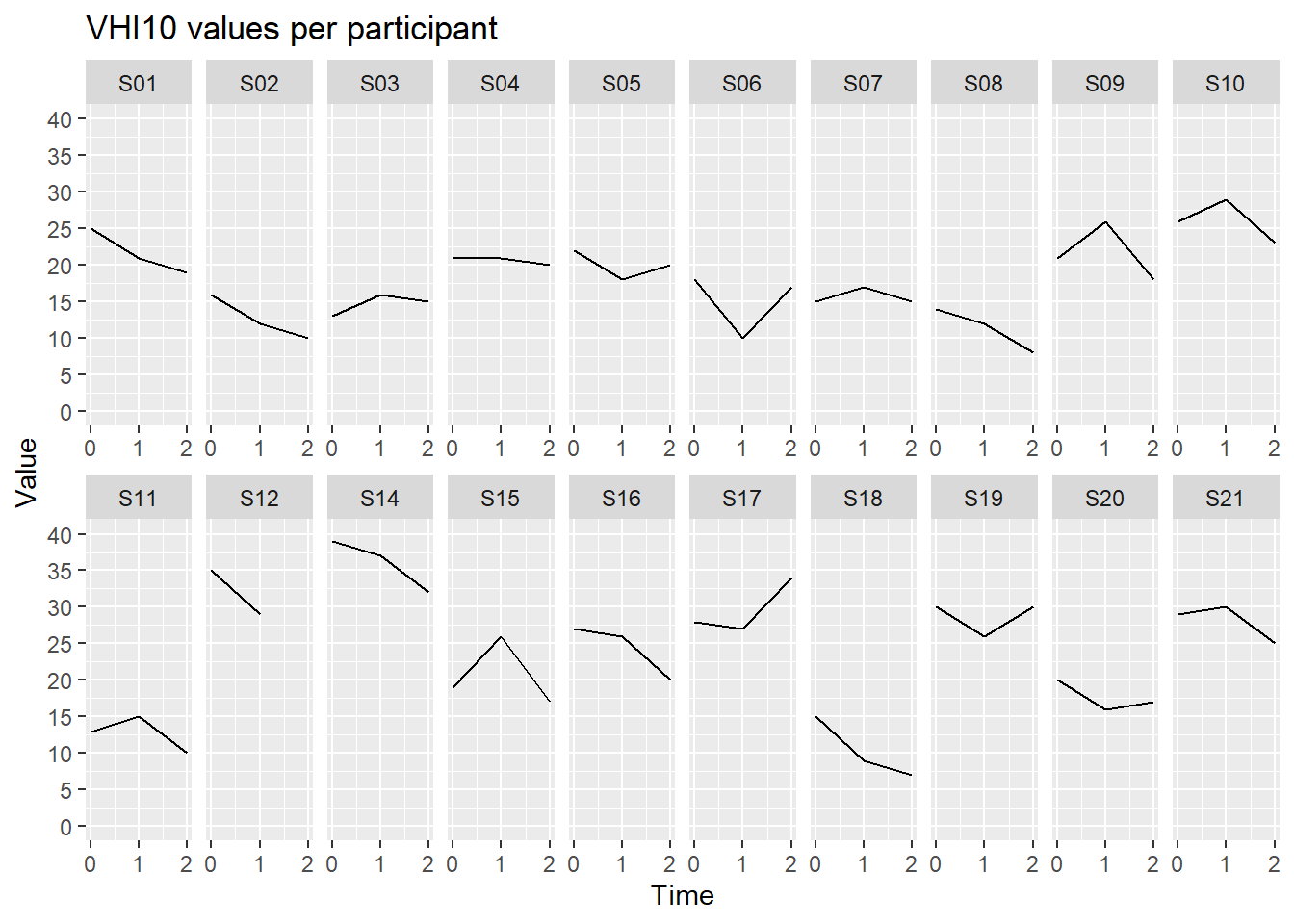

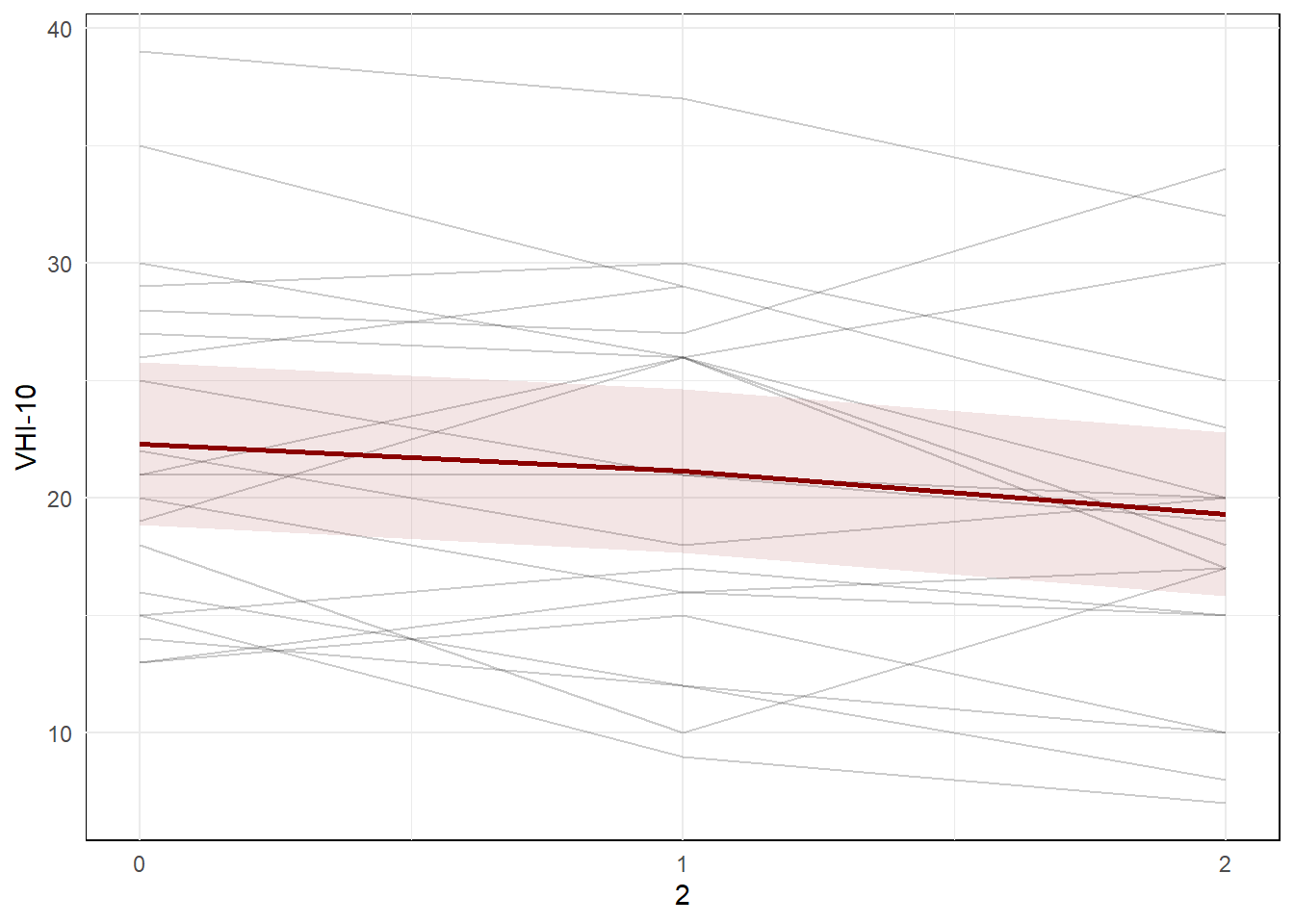


**
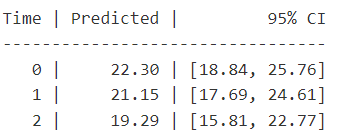
**

## Appendix 8: LME Model of the PROM: V-RQOL

**
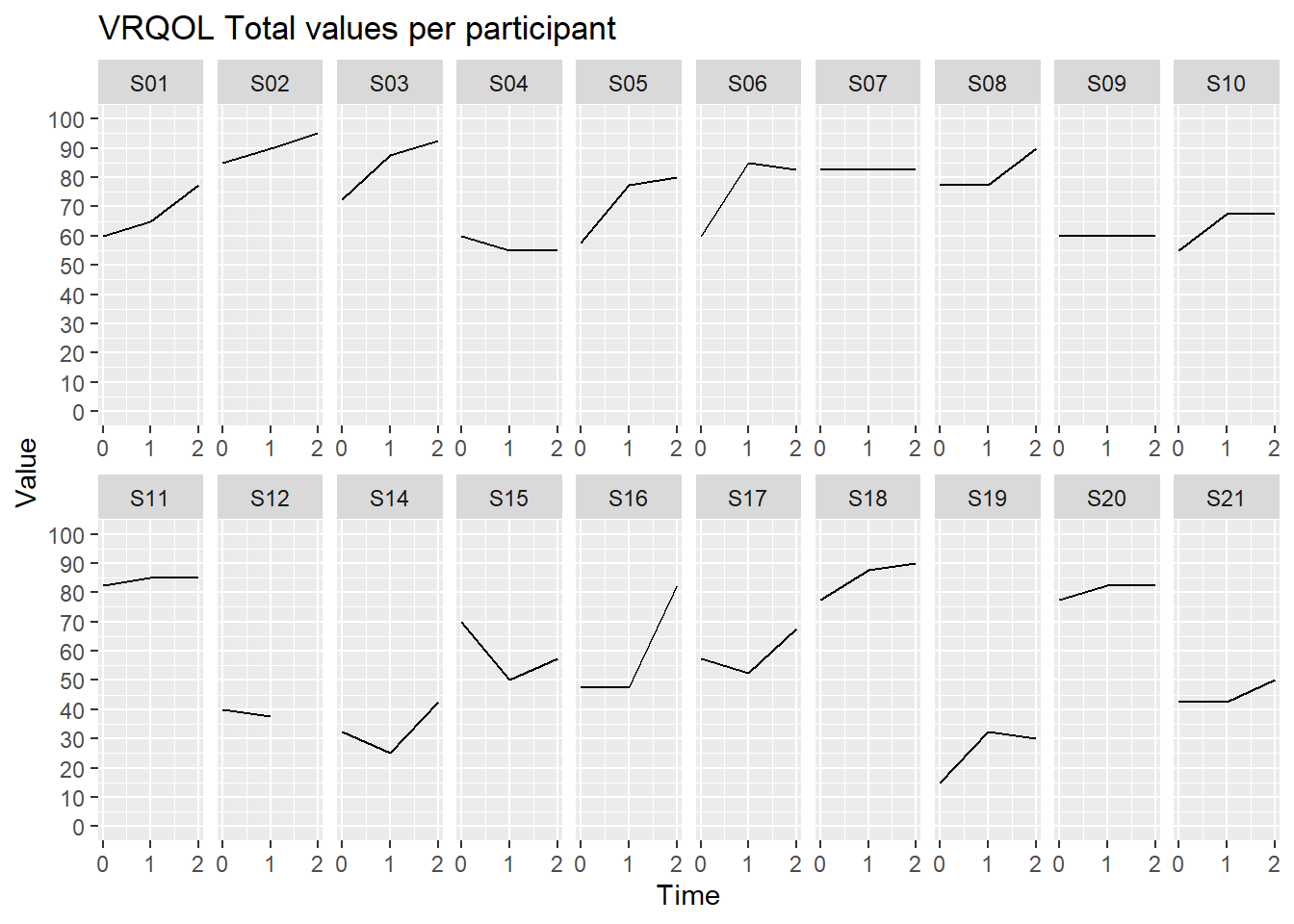
**

**
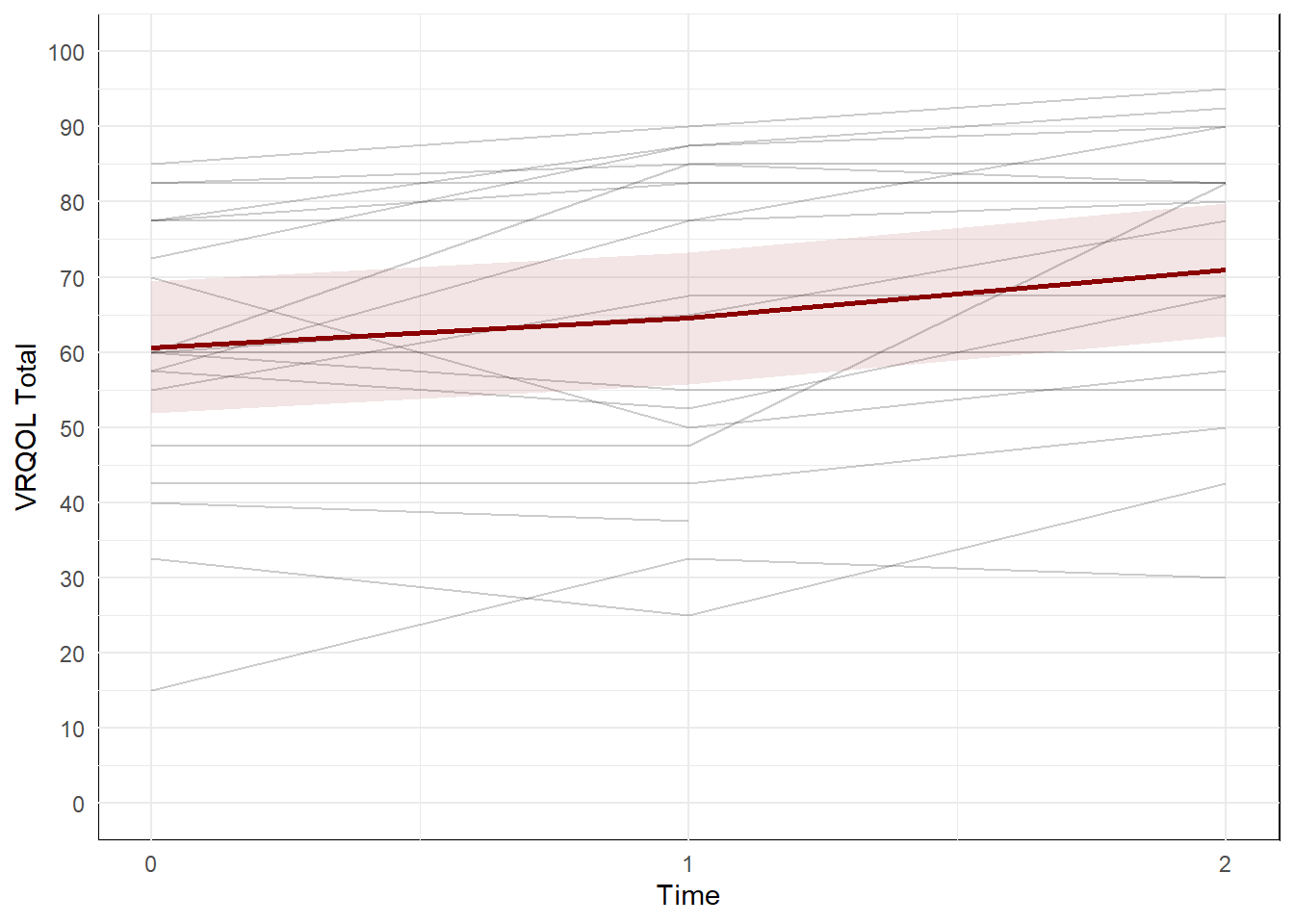
**


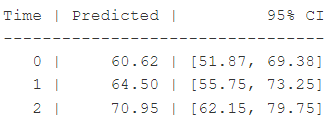


**
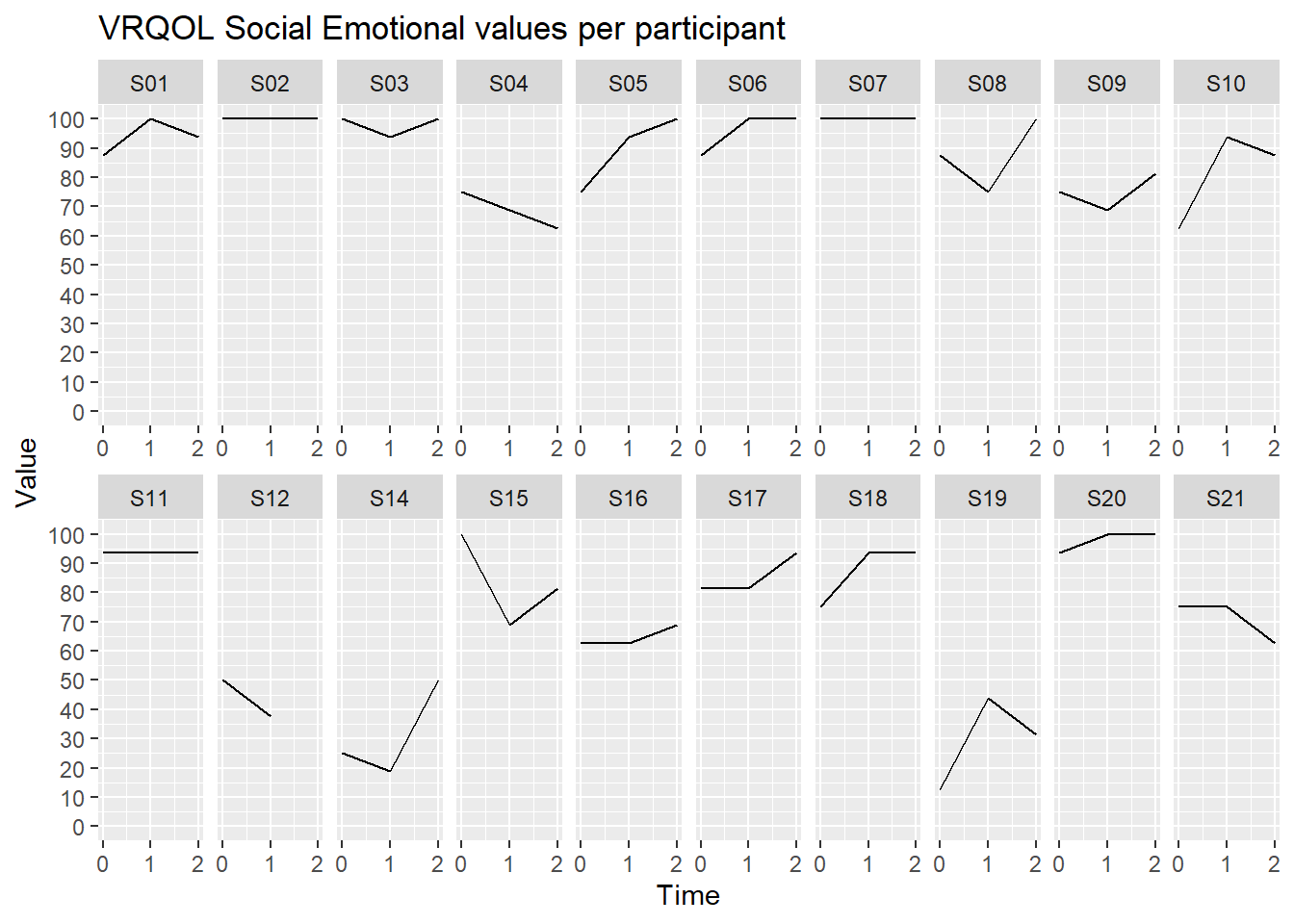
**

**
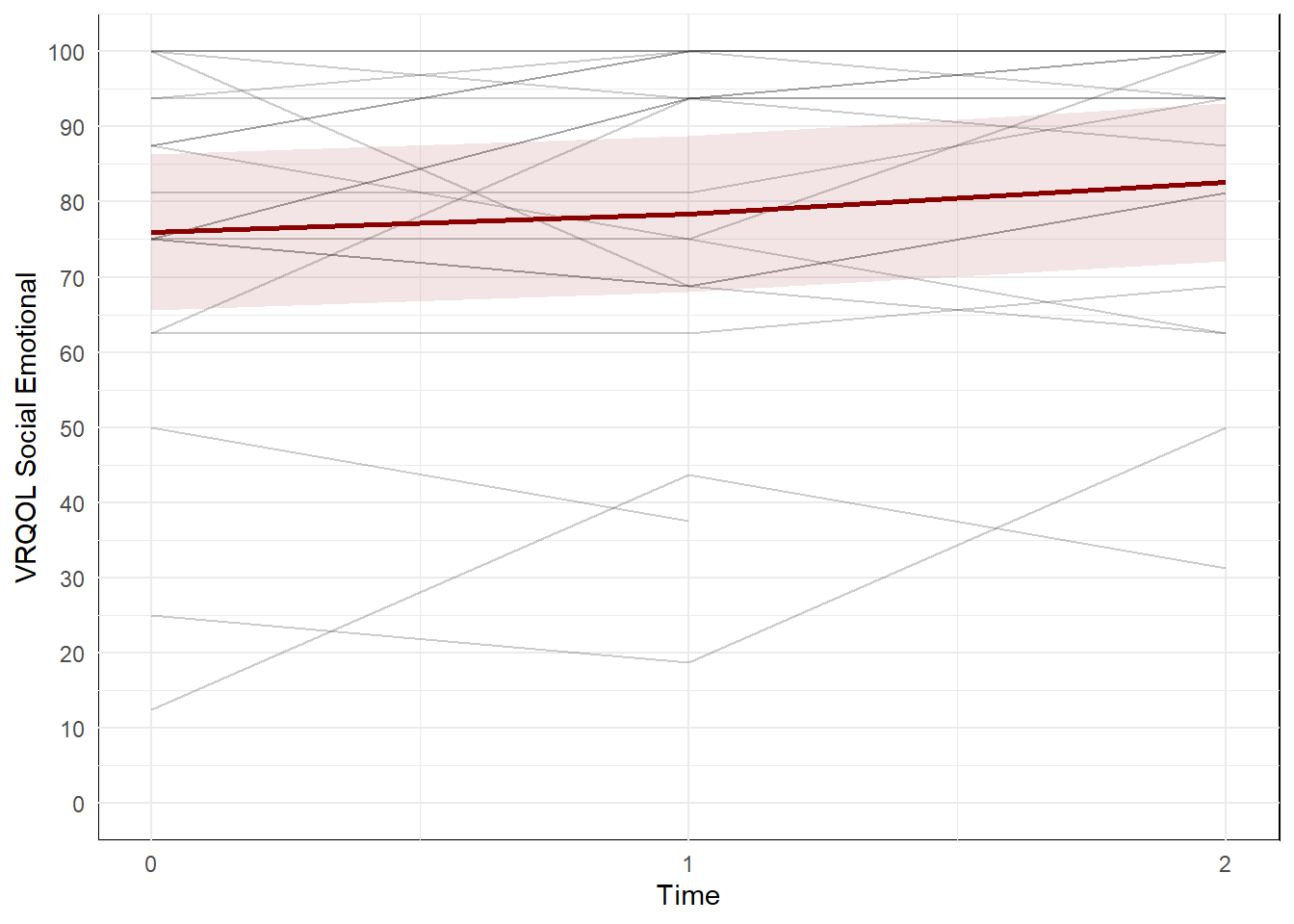
**


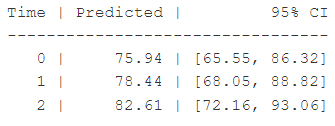


**
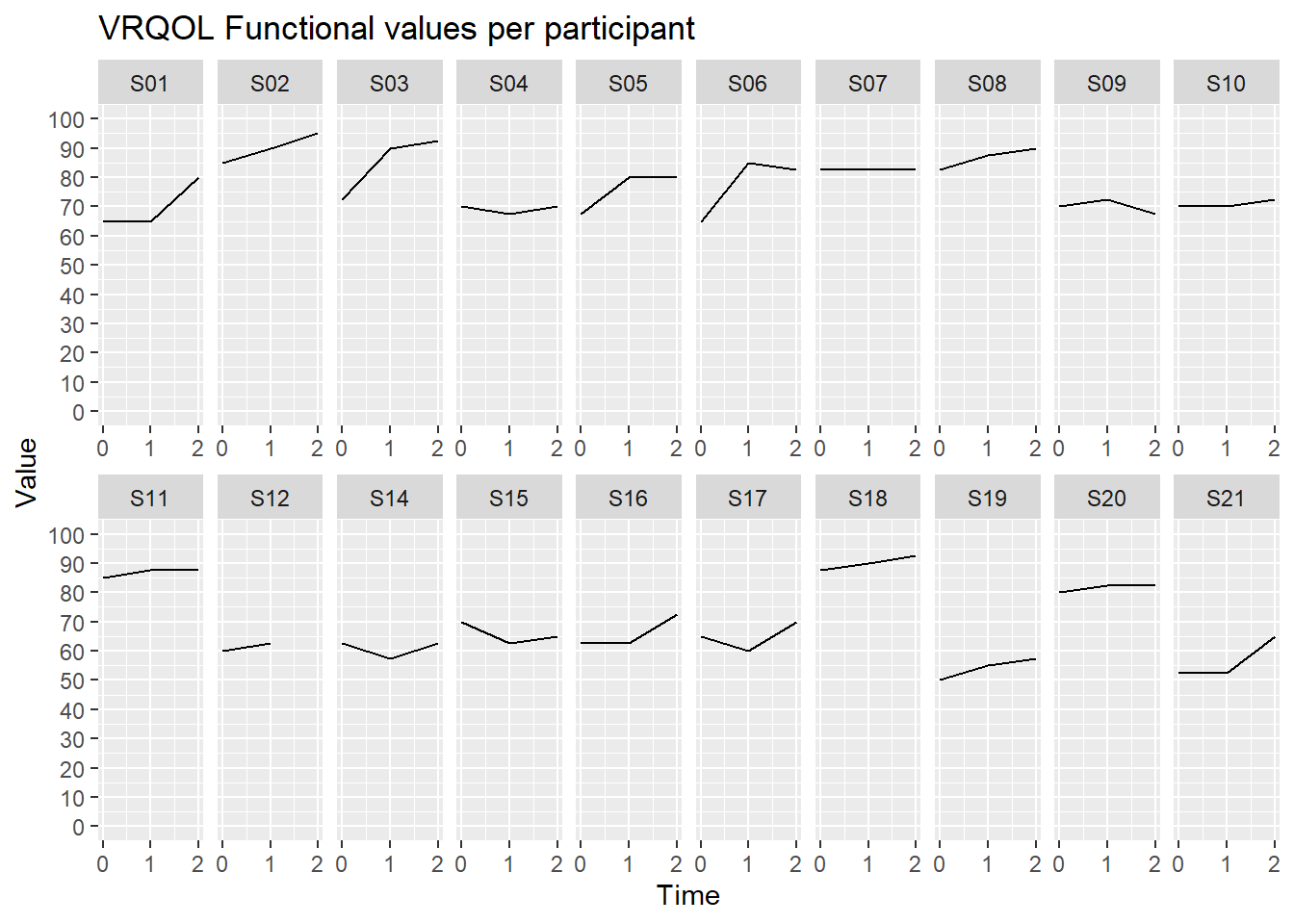
**

**
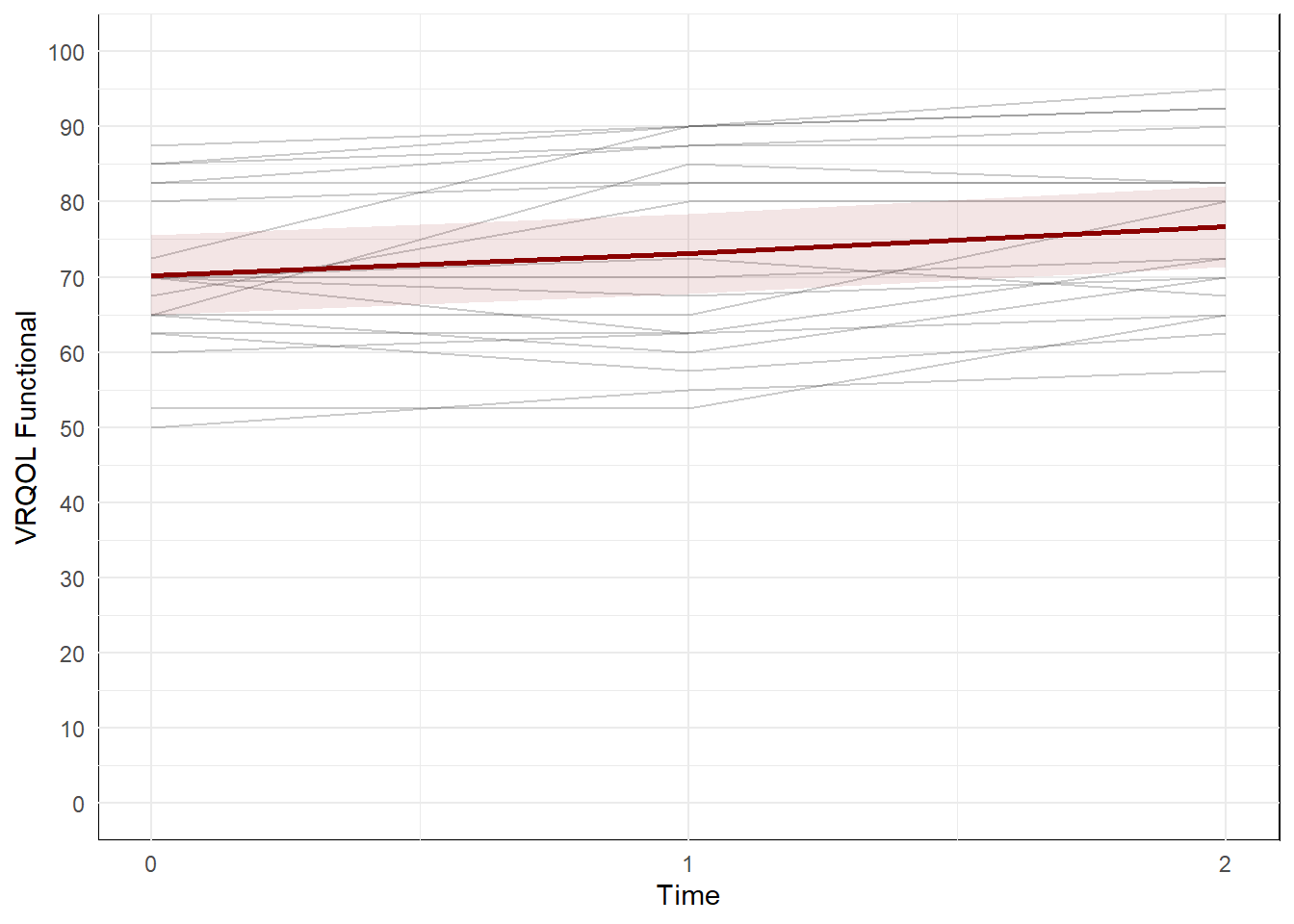
**


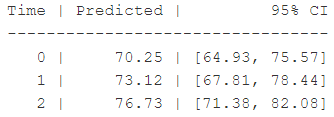


## Appendix 9: LME Model of the PROM: CPIB-10

**
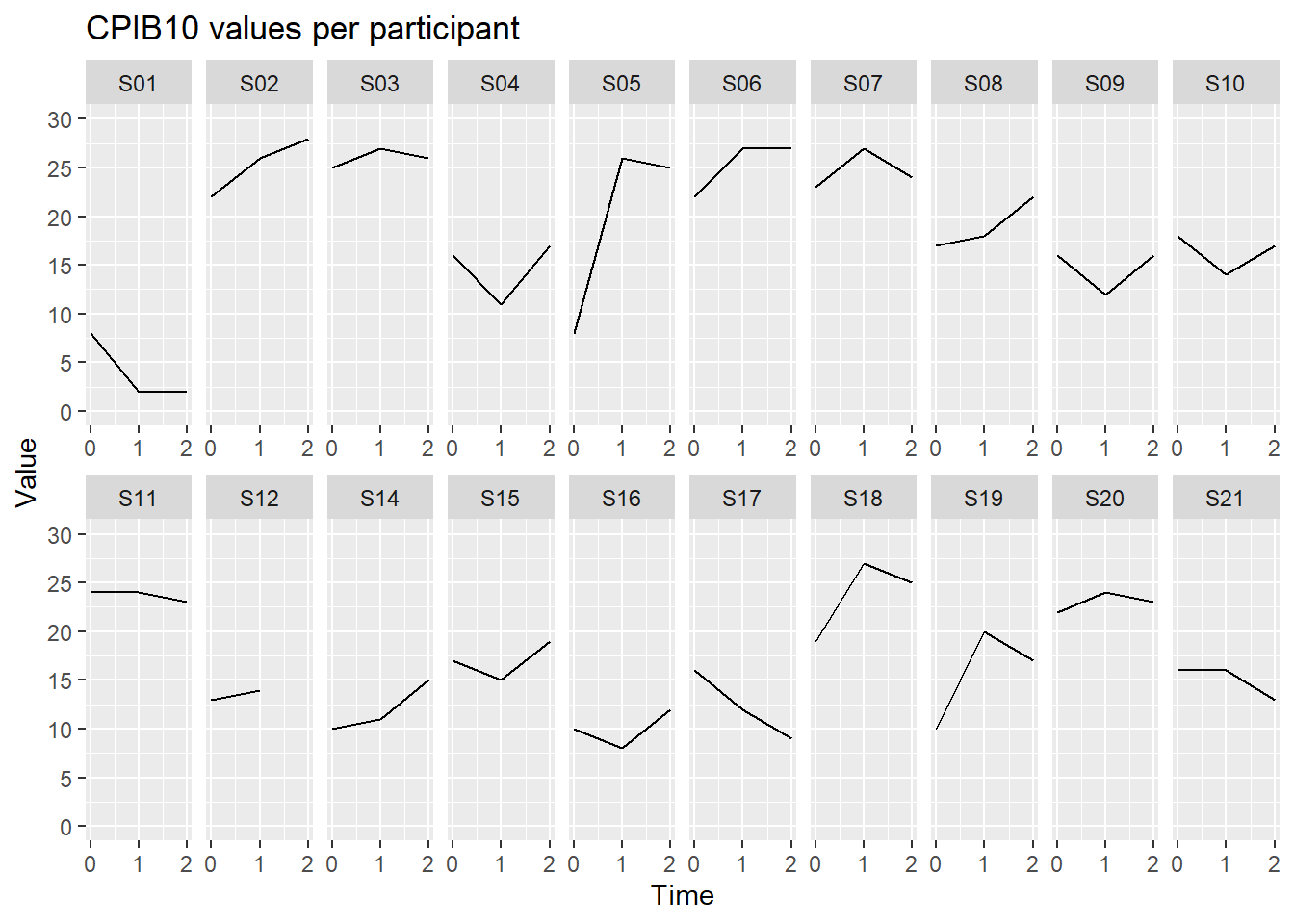
**

**
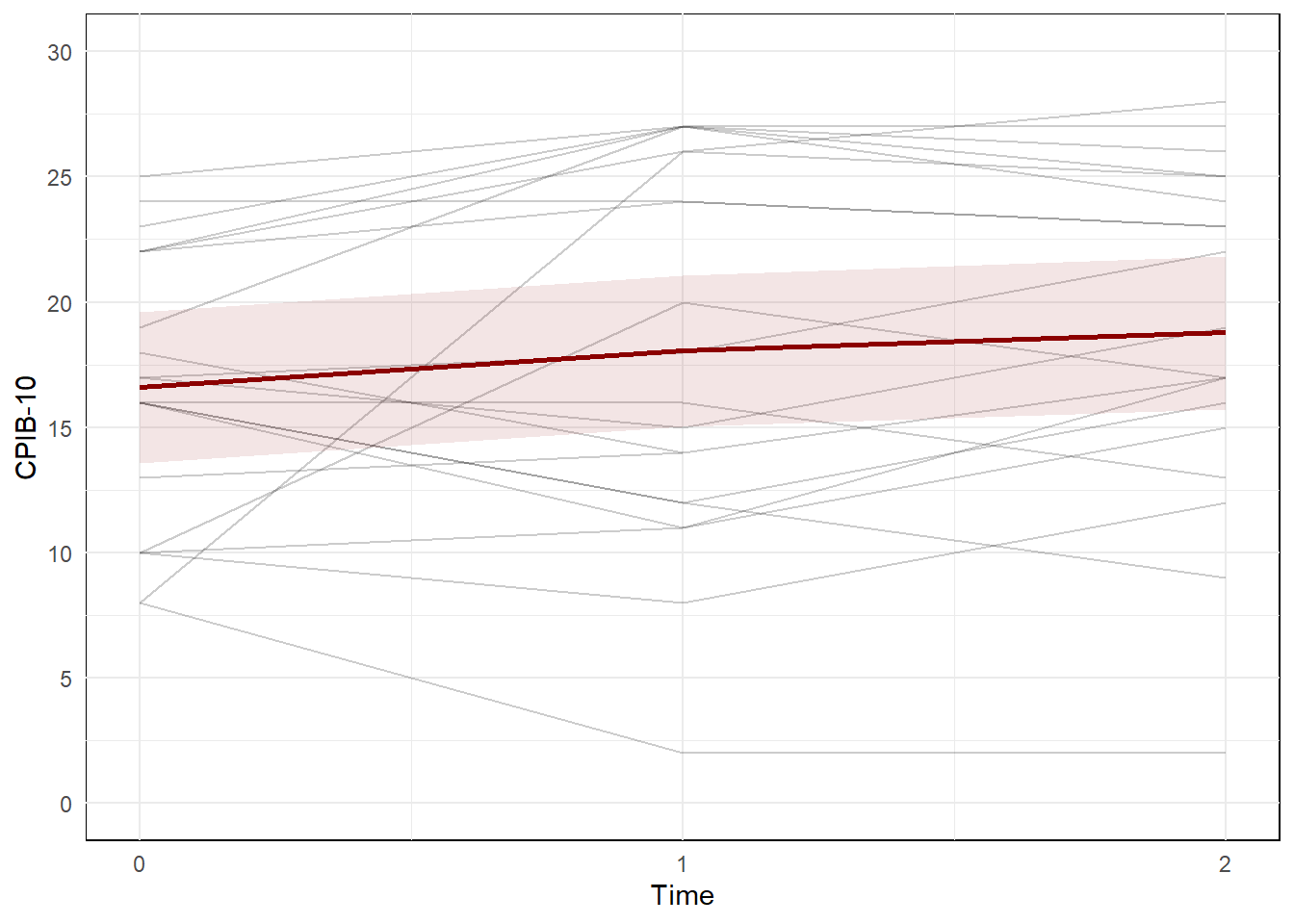
**

**
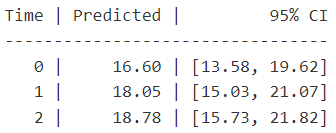
**

## Appendix 10: Overview of raw data of secondary outcomes

Table 1 Raw data on secundary outcomes at participant level across Time Points (T0, T1 and T2).

| **Secondary outcomes measured in mmHg** | | | | | | | | | | | | | | | | | | | | | | **x̄ (range)** |
| --- | --- | --- | --- | --- | --- | --- | --- | --- | --- | --- | --- | --- | --- | --- | --- | --- | --- | --- | --- | --- | --- | --- |
| **Participants** | | **S01** | **S02^#^** | **S03^#^** | **S04^#^** | **S05** | **S06** | **S07** | **S08** | **S09^#^** | **S10** | **S11** | **S12** | **S14^#^** | **S15** | **S16** | **S17** | **S18** | **S19^#^** | **S20** | **S21** |  |
|  | | | | | | | | | | | | | | | | | | | | | | |
| **T0** | SVp.mean | 32 | 37 | 27 | 44 | 28 | 18 | 26 | 27 | 32 | 14 | 22 | 43 | 9 | 22 | 17 | 21 | 21 | 26 | 28 | 50 | 27 (9-50) |
|  | SVp.max | 39 | 42 | 31 | 46 | 31 | 19 | 30 | 43 | 37 | 15 | 23 | 50 | 15 | 26 | 18 | 23 | 22 | 32 | 32 | 56 | 31 (15-56) |
|  | CSp.mean | 8 | 26 | 20 | 33 | 15 | 36 | 20 | 22 | 21 | 23 | 27 | 19 | 12 | 22 | 9 | 23 | 7 | 35 | 18 | 26 | 21 (7-36) |
|  | CSp.max | 23 | 42 | 36 | 66 | 25 | 46 | 25 | 37 | 35 | 29 | 38 | 50 | 13 | 28 | 24 | 28 | 20 | 41 | 24 | 50 | 34 (13-66) |
|  | HRIM neutral | 24 | 583 | 534 | 1009 | x | 368 | 848 | 213 | 430 | 424 | 324 | 318 | x | 125 | 141 | 186 | 512 | x | 143 | x | 386 (24-1009) |
|  | HRIM soft | 87 | 149 | 317 | 810 | x | 144 | 603 | 128 | 196 | 446 | 373 | 114 | x | 77 | 116 | 144 | 484 | x | 44 | x | 265 (44-810) |
|  | HRIM low | 286 | 388 | 467 | 225 | x | 211 | 765 | 117 | 297 | 391 | 296 | 142 | x | 121 | 178 | 180 | 458 | x | 154 | x | 292 (117-765) |
|  | HRIM high | 96 | 519 | 1034 | 785 | x | 308 | 796 | 377 | 344 | 582 | 469 | 254 | x | 182 | 180 | 348 | 951 | x | 123 | x | 459 (96-1034) |
|  | HRIM loud | 231 | 666 | 930 | 2395 | x | 652 | 1761 | 702 | 836 | 676 | 376 | 228 | x | 310 | 118 | 354 | 1100 | x | 156 | x | 718 (118-2395) |
|  | | | | | | | | | | | | | | | | | | | | | | |
| **T1** | SVp.mean | 37 | 46 | 21 | 35 | 15 | 25 | 44 | 58 | 20 | 20 | 30 | 38 | 8 | 22 | 13 | 27 | 18 | 28 | 18 | 36 | 28 (8-58) |
|  | SVp.max | 44 | 53 | 24 | 39 | 20 | 30 | 49 | 66 | 23 | 22 | 32 | 42 | 10 | 23 | 17 | 30 | 20 | 30 | 22 | 39 | 32 (10-66) |
|  | CSp.mean | 7 | 19 | 16 | 37 | 14 | 48 | 18 | 30 | 19 | 26 | 25 | 8 | 6 | 22 | 21 | 20 | 16 | 33 | 17 | 10 | 21 (6-48) |
|  | CSp.max | 16 | 24 | 30 | 57 | 20 | 59 | 30 | 59 | 26 | 31 | 38 | 34 | 9 | 29 | 25 | 52 | 37 | 44 | 22 | 29 | 34 (9-59) |
|  | HRIM neutral | 136 | 181 | 997 | 444 | 122 | 622 | 411 | 335 | 389 | 565 | 289 | 221 | x | 257 | 173 | 163 | x | x | 564 | x | 367 (122-997) |
|  | HRIM soft | 34 | 20 | 242 | 355 | 154 | 190 | 351 | 159 | 138 | 364 | 137 | 172 | x | 89 | 197 | 178 | x | x | 187 | x | 185 (20-364) |
|  | HRIM low | 90 | 33 | 856 | 237 | 219 | 209 | 353 | 189 | 288 | 461 | 229 | 146 | x | 99 | 243 | 195 | x | x | 183 | x | 252 (33-856) |
|  | HRIM high | 291 | 99 | 386 | 408 | 201 | 138 | 542 | 369 | 368 | 454 | 395 | 218 | x | 152 | 425 | 196 | x | x | 674 | x | 332 (99-674) |
|  | HRIM loud | 211 | 289 | 871 | 612 | 296 | 862 | 498 | 1178 | 380 | 755 | 712 | 299 | x | 378 | 564 | 247 | x | x | 695 | x | 553 (211-1178) |
|  | | | | | | | | | | | | | | | | | | | | | | |
| **T2** | SVp.mean | 26 | 24 | 28 | 29 | 12 | 20 | 23 | 40 | 31 | 16 | 17 | x | x | 33 | 17 | 34 | 24 | 21 | 17 | 34 | 25 (12-40) |
|  | SVp.max | 29 | 26 | 30 | 29 | 13 | 21 | 34 | 42 | 38 | 17 | 19 | x | x | 34 | 19 | 44 | 25 | 30 | 20 | 42 | 28 (13-44) |
|  | CSp.mean | 8 | 23 | 18 | 35 | 10 | 26 | 15 | 36 | 24 | 18 | 24 | x | 13 | 23 | 20 | 41 | 17 | 20 | 19 | 20 | 22 (8-41) |
|  | CSp.max | 23 | 29 | 31 | 45 | 19 | 36 | 22 | 50 | 28 | 21 | 43 | x | 14 | 37 | 25 | 59 | 24 | 42 | 32 | 37 | 32 (14-59) |
|  | HRIM neutral | 137 | 244 | 539 | 853 | 104 | 624 | 365 | 233 | 1095 | 408 | 292 | x | 1431 | 121 | 144 | 559 | 735 | x | 258 | x | 479 (104-1431) |
|  | HRIM soft | 96 | 61 | 168 | 243 | 63 | 288 | 171 | 190 | 320 | 305 | 259 | x | 204 | 83 | 101 | 362 | 375 | x | 94 | x | 199 (61-375) |
|  | HRIM low | 343 | 682 | 234 | 215 | x | 299 | 156 | 188 | 439 | 367 | 280 | x | 225 | 103 | 144 | 324 | 403 | x | 324 | x | 295 (103-682) |
|  | HRIM high | 267 | x | 418 | 334 | 137 | 443 | 390 | 369 | 852 | 542 | 462 | x | 380 | 182 | 275 | 364 | 970 | x | 401 | x | 424 (137-970) |
|  | HRIM loud | 340 | 1040 | 701 | 875 | 381 | 1202 | 634 | 698 | 1079 | 681 | 485 | x | 495 | 458 | 267 | 668 | 1351 | x | 429 | x | 693 (267-1351) |

**Abbreviations**: **^#^** participants with a history of stenosis and dilatations; SVp.mean, mean tracheal pressure during sustained vowel; SVp.max, maximal tracheal pressure during sustained vowel; CSp.mean, mean tracheal pressure during continued speech; CSp.max, maximal tracheal pressure during continuous speech; HRIM, High-Resolution Impedance Manometry of sustained vowel on different extremities (neutral, soft, low, high and loud).

## Appendix 11: LME model on maximum tracheal pressure (mmHg) of sustained vowel /a/

**
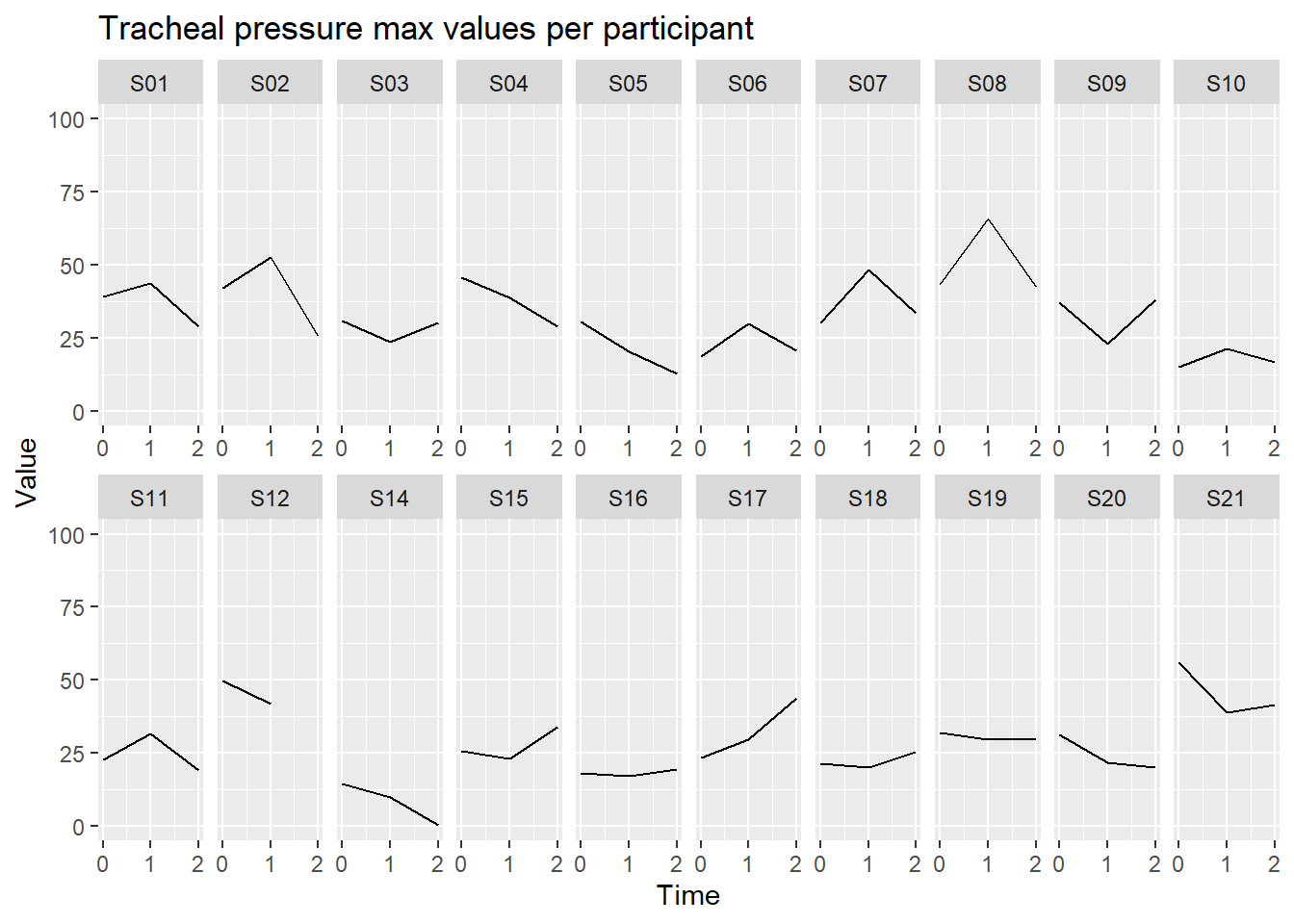
**

**
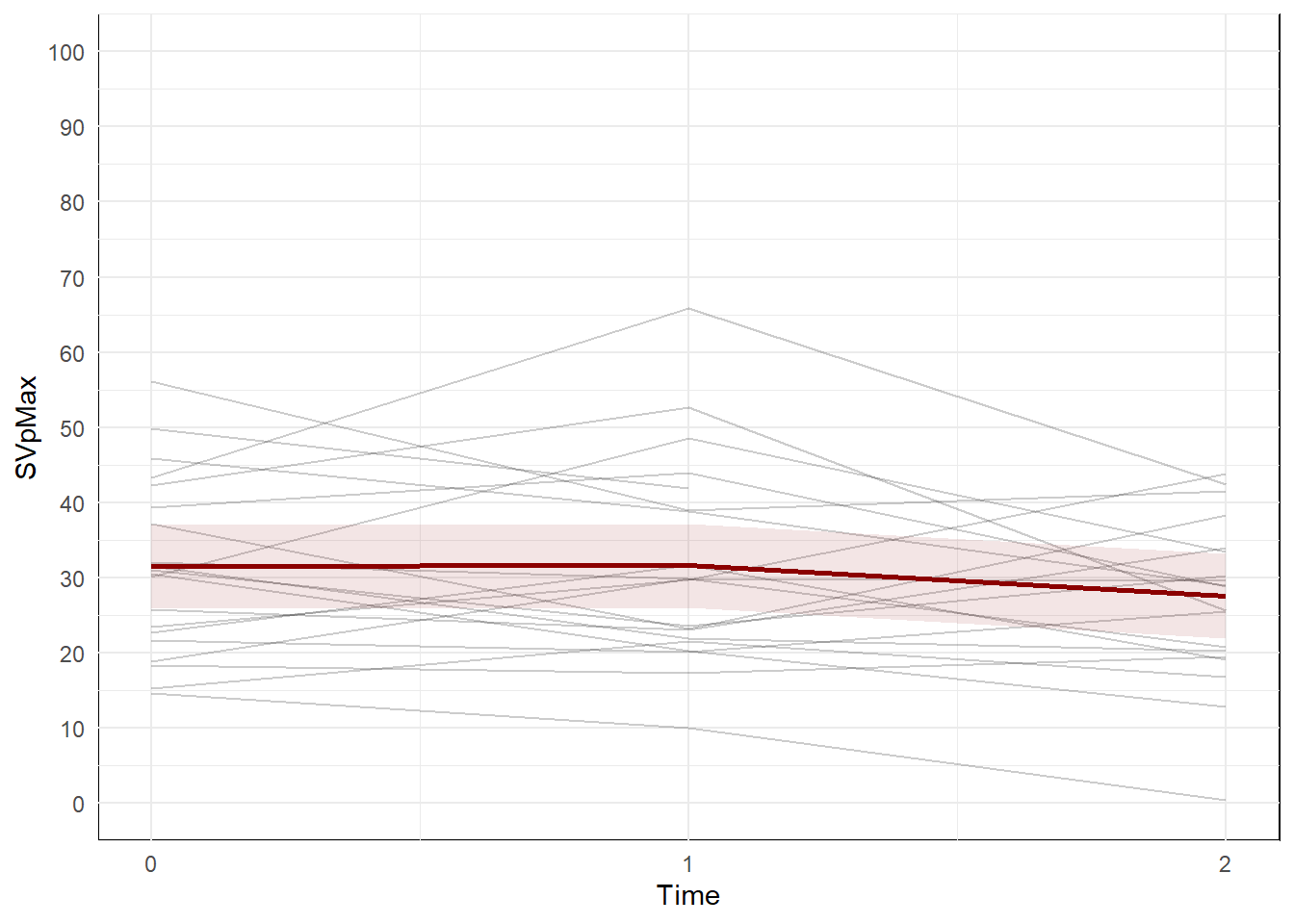
**

**
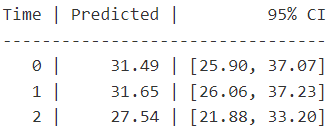
**

## Appendix 12: LME model on mean tracheal pressure (mmHg) of sustained vowel /a/

**
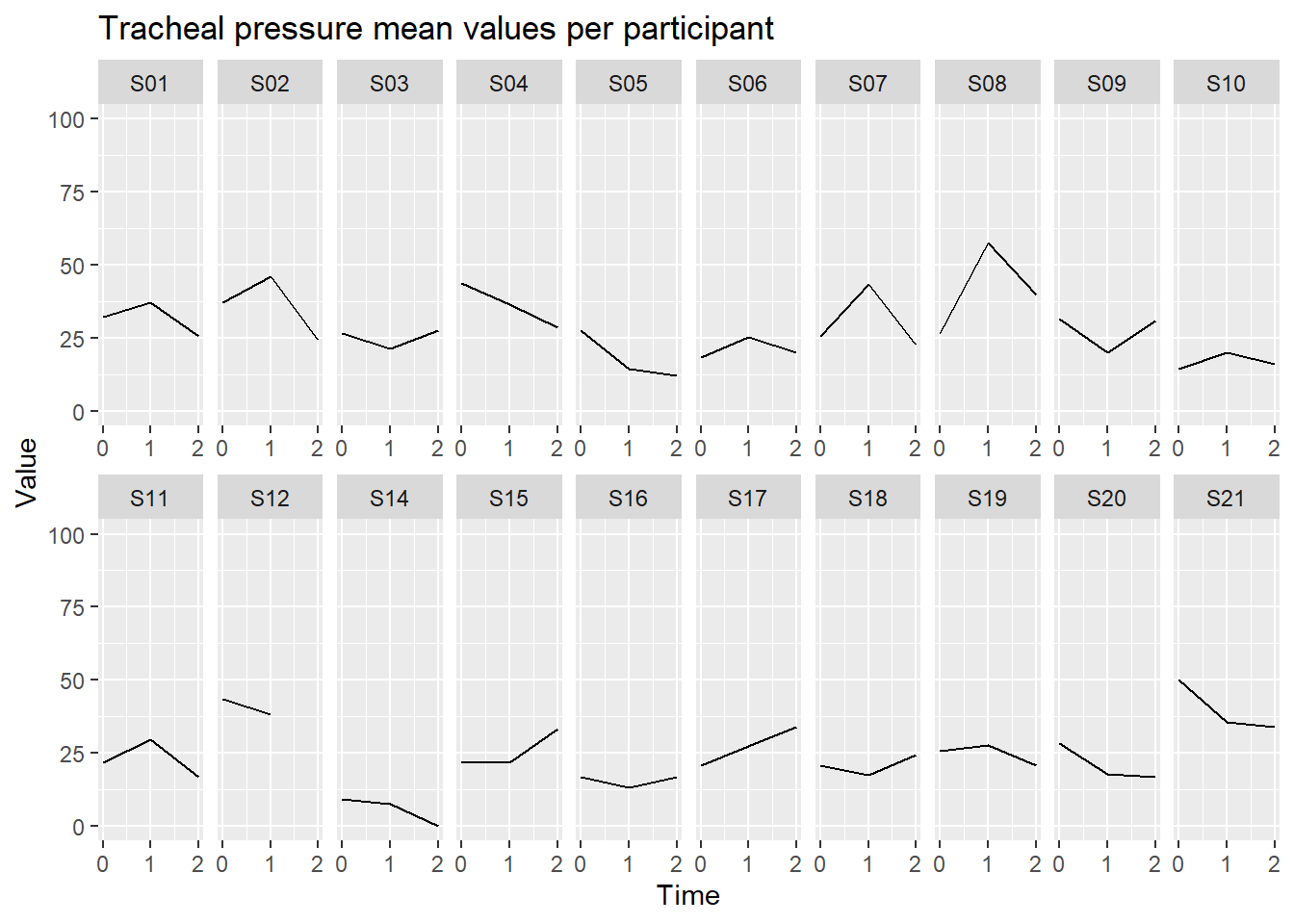
**

**
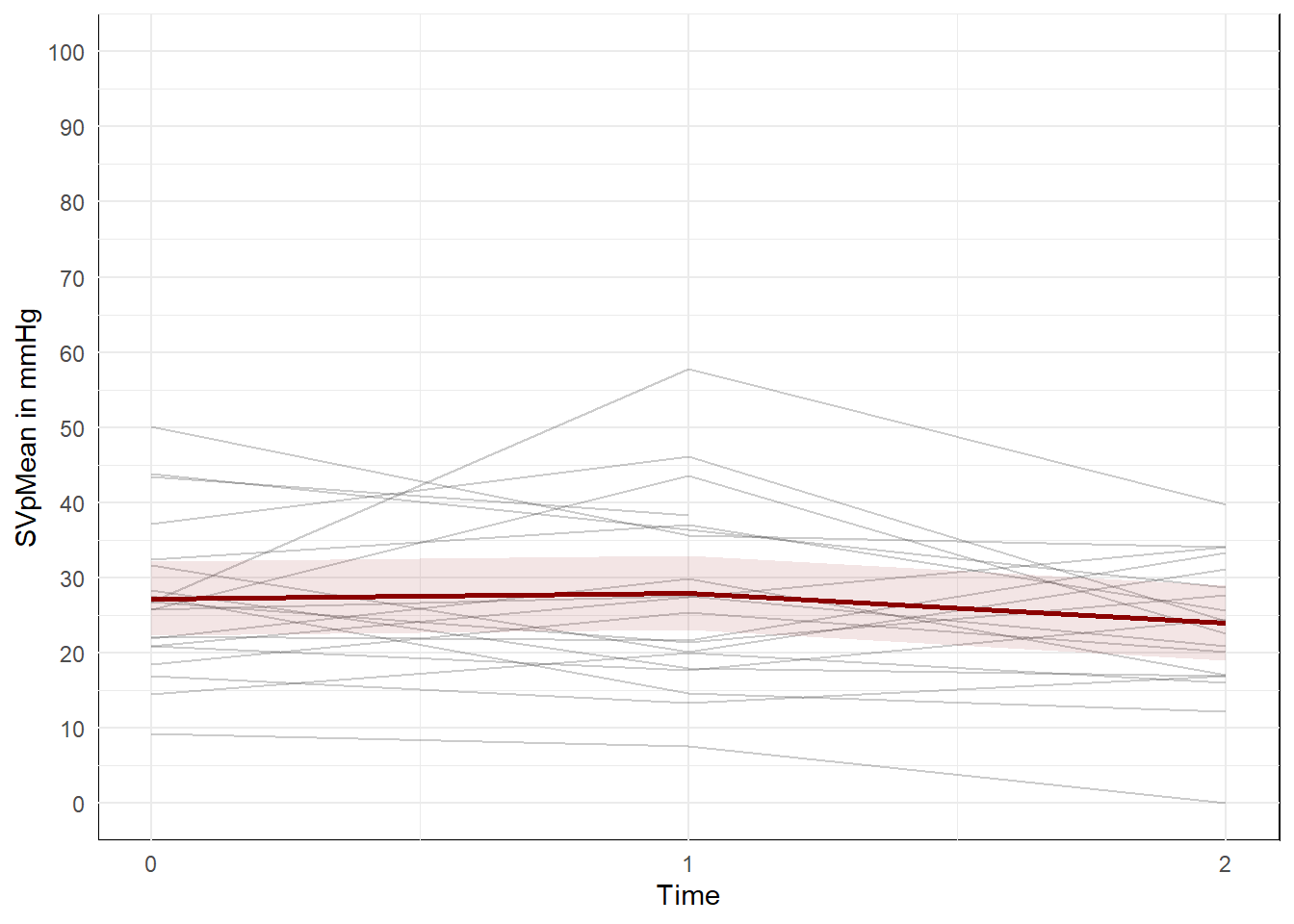
**

**
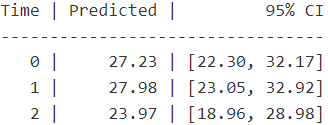
**

## Appendix 13: LME model on maximum tracheal pressure during (mmHg) of continuous speech

**
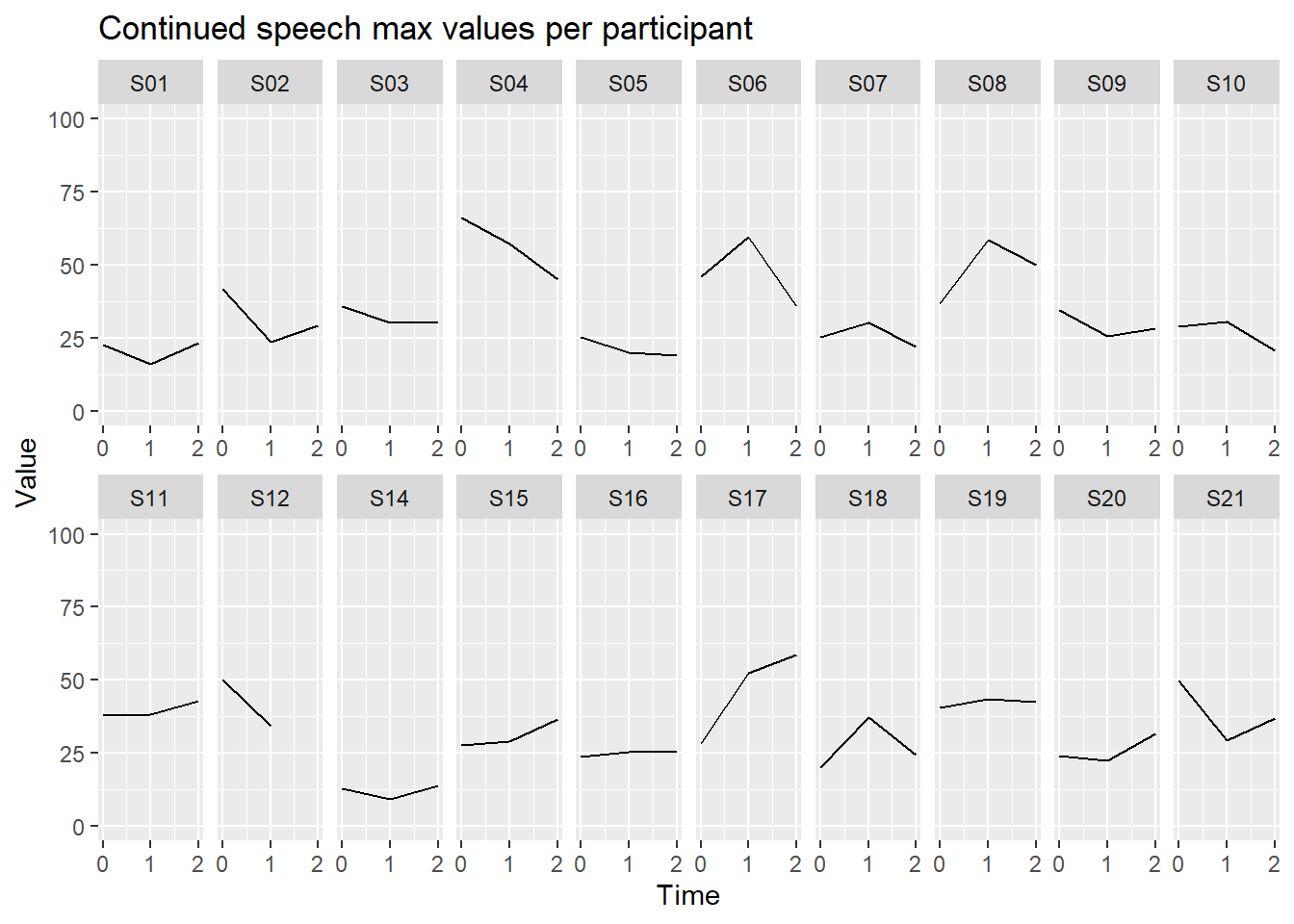

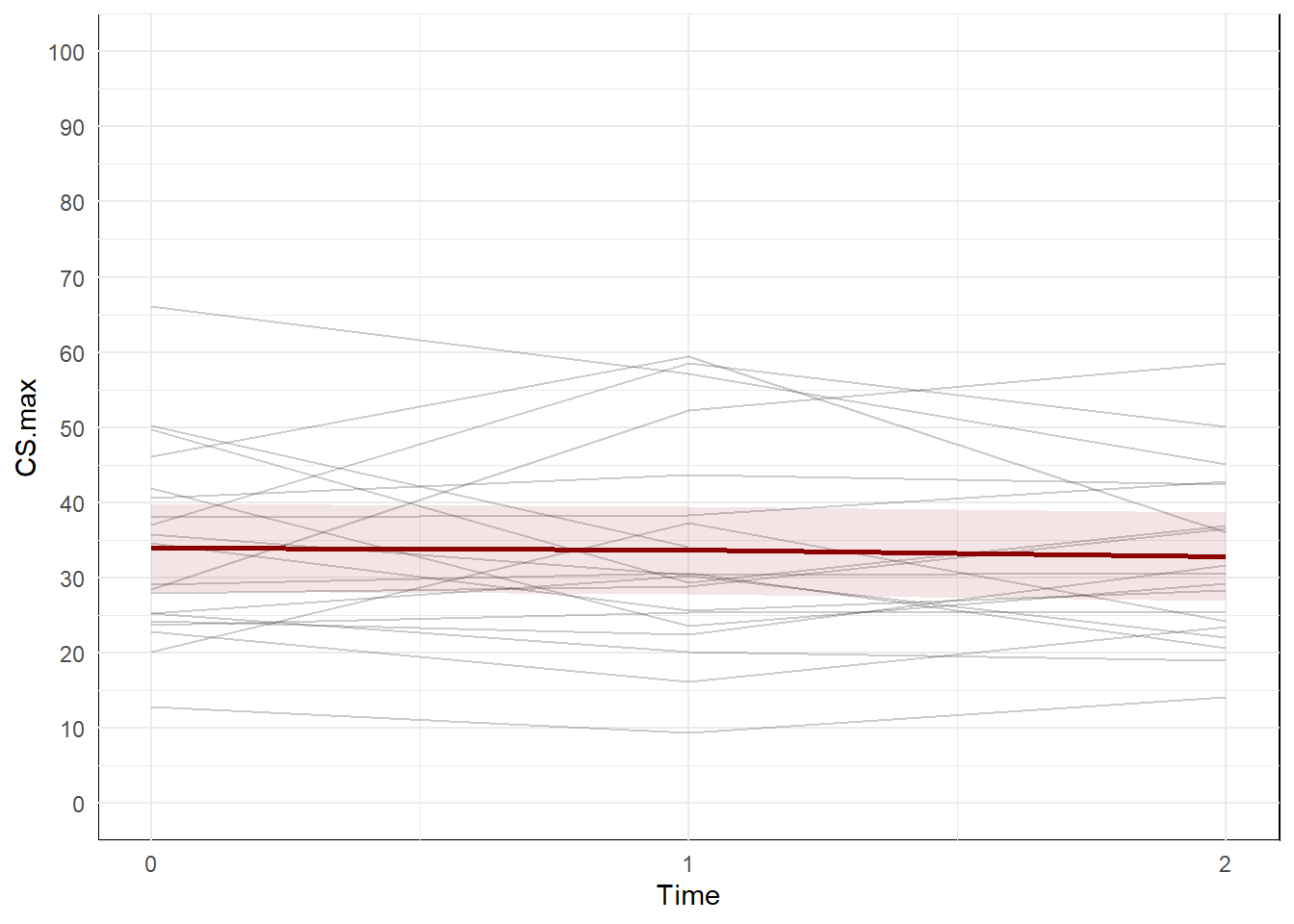

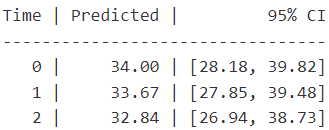
**

## Appendix 14: LME model on mean tracheal pressure (mmHg) during continuous speech

**
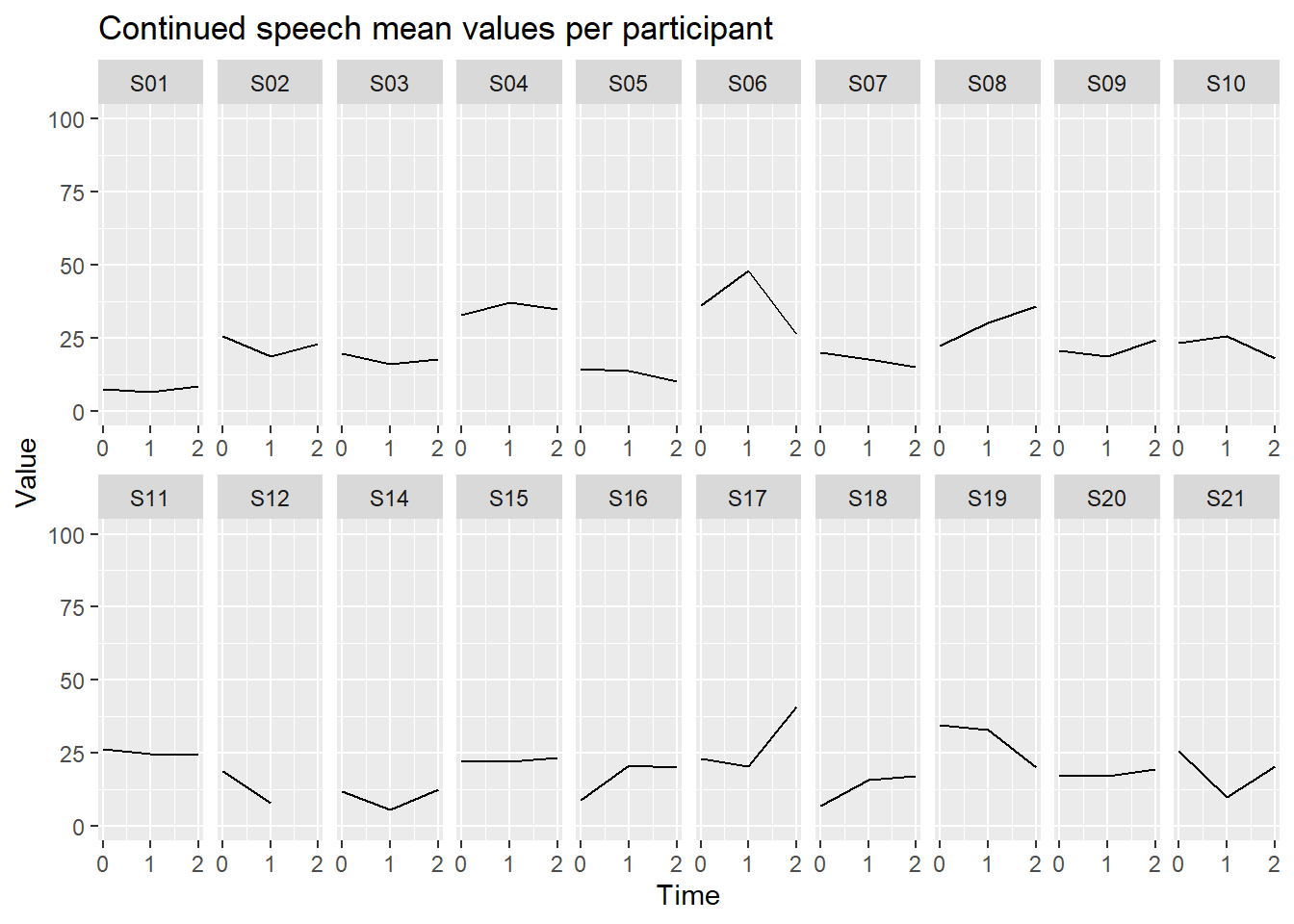

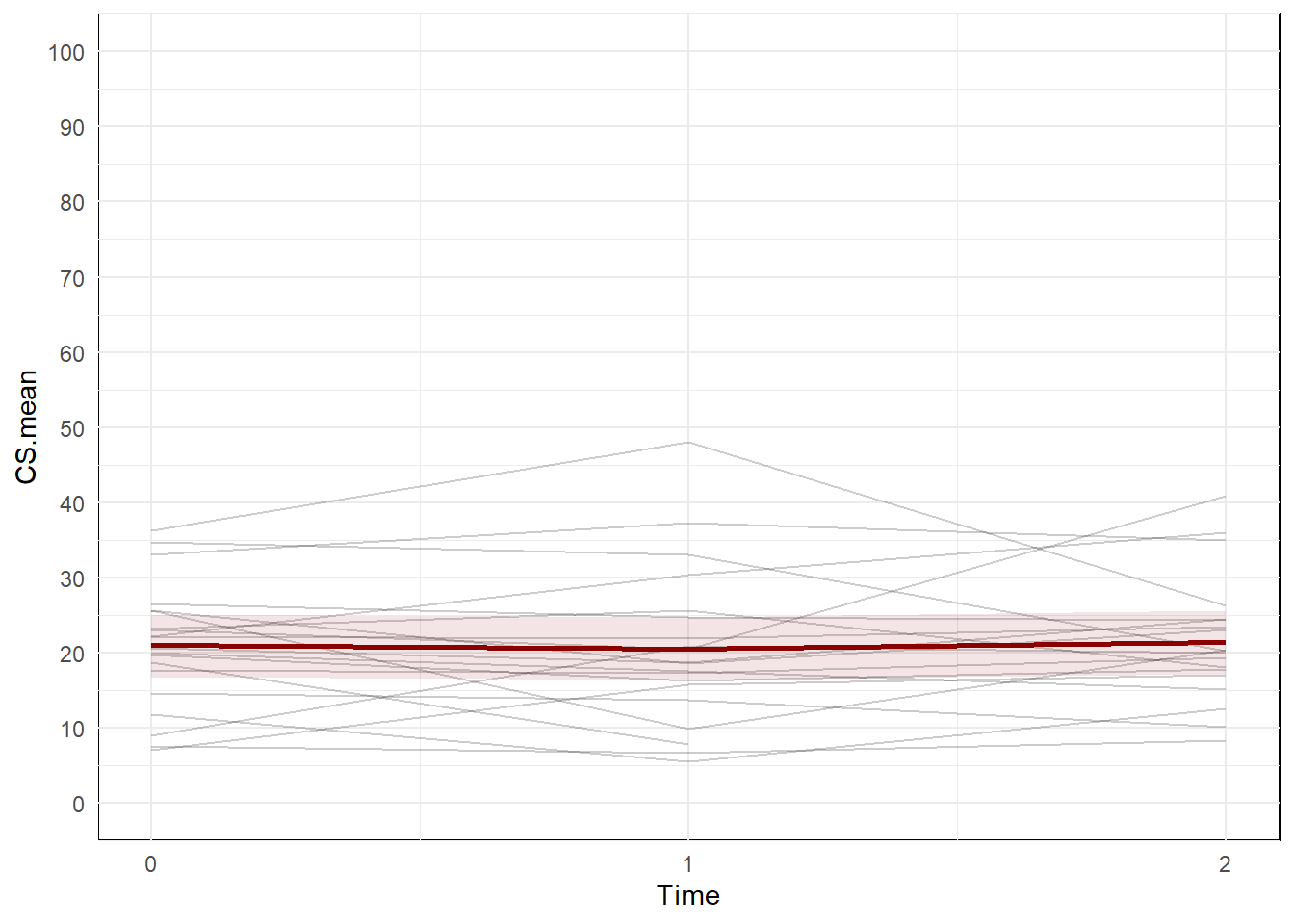
**

## **
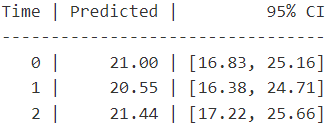
**

## Appendix 15: LME model on esophageal pressure (in mmHg) of a neutral sustained vowel /a/

**
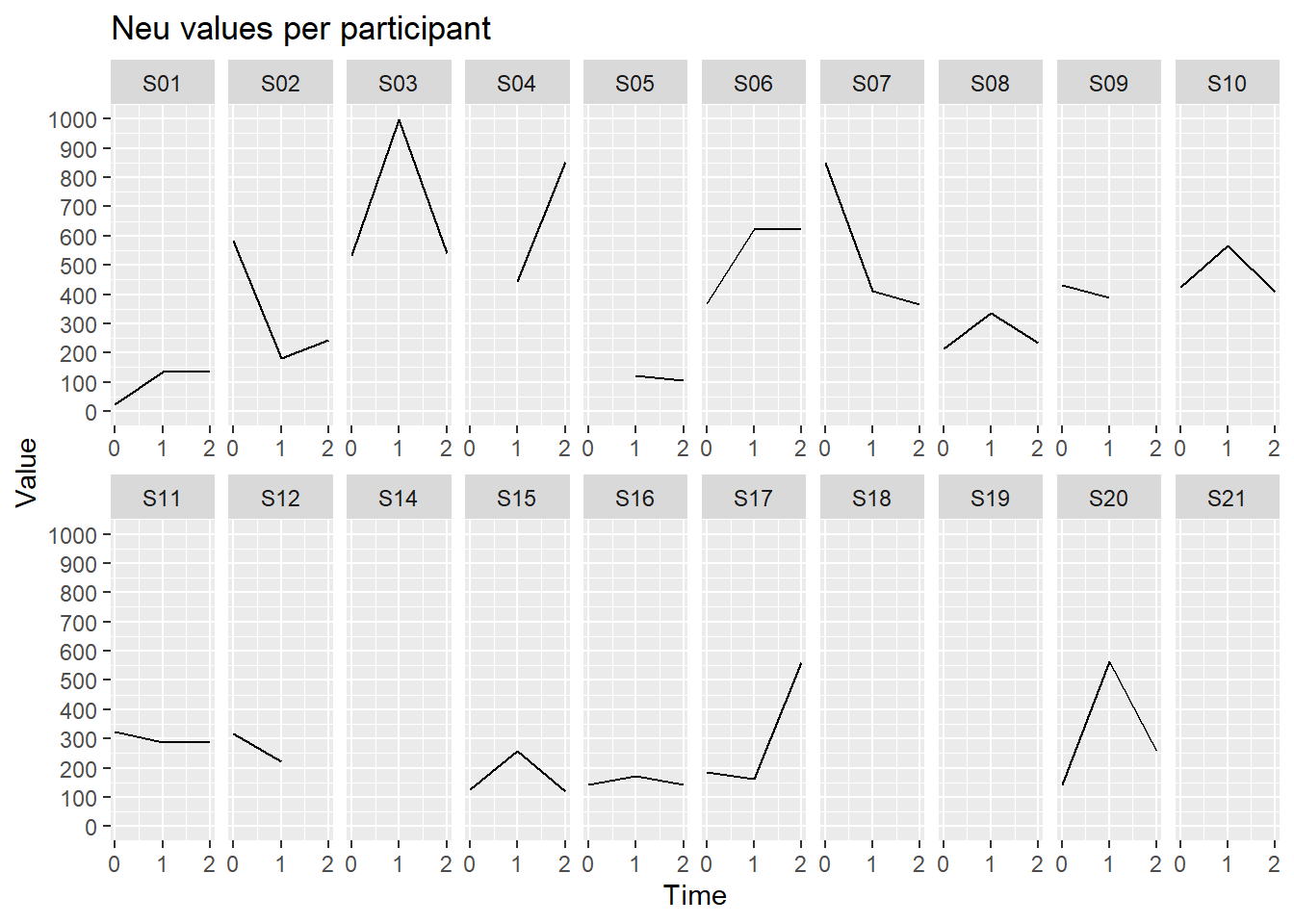
**

**
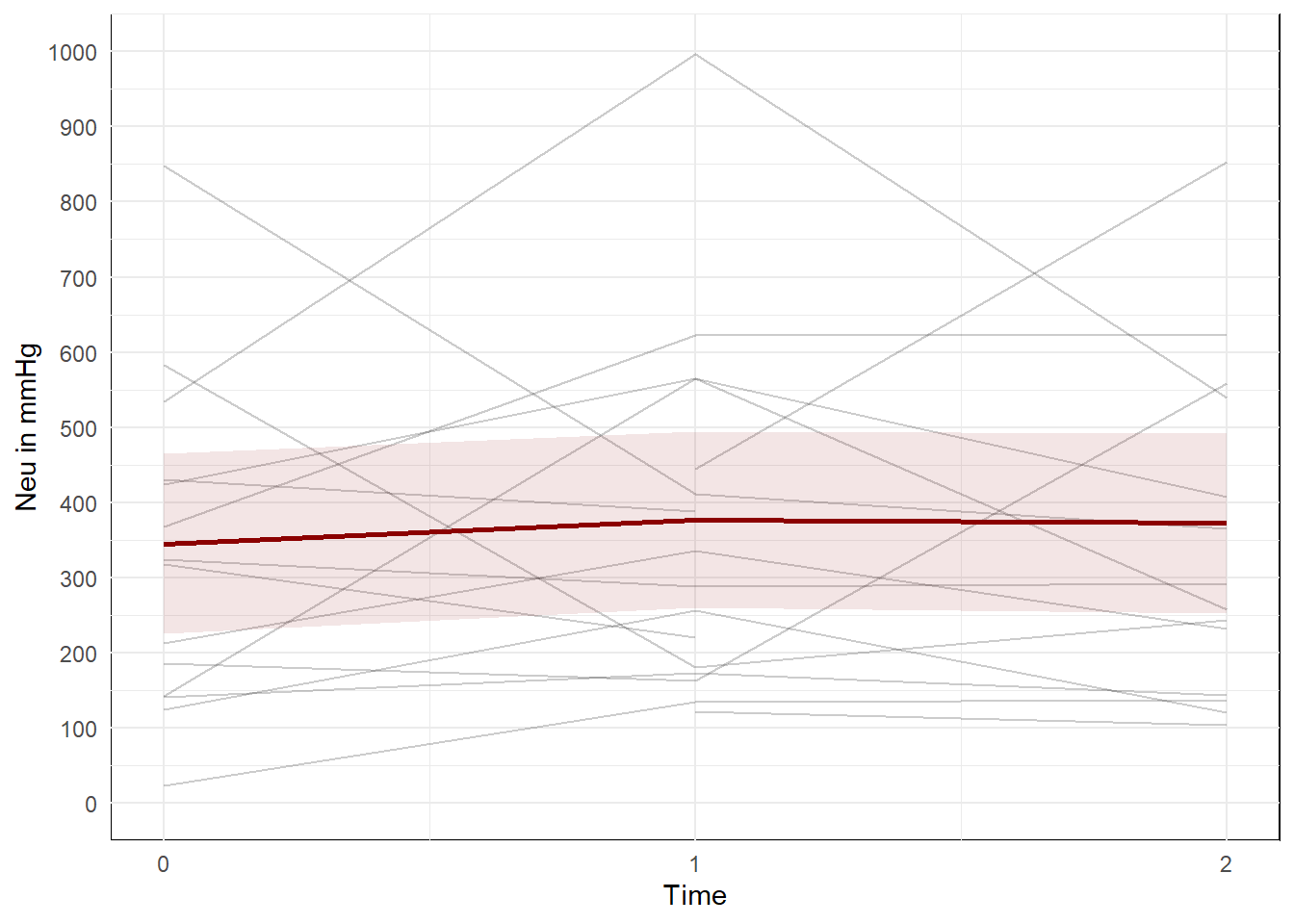
**

**
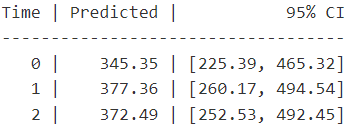
**

## Appendix 16: LME model on esophageal pressure (in mmHg) of a soft sustained vowel /a/

**
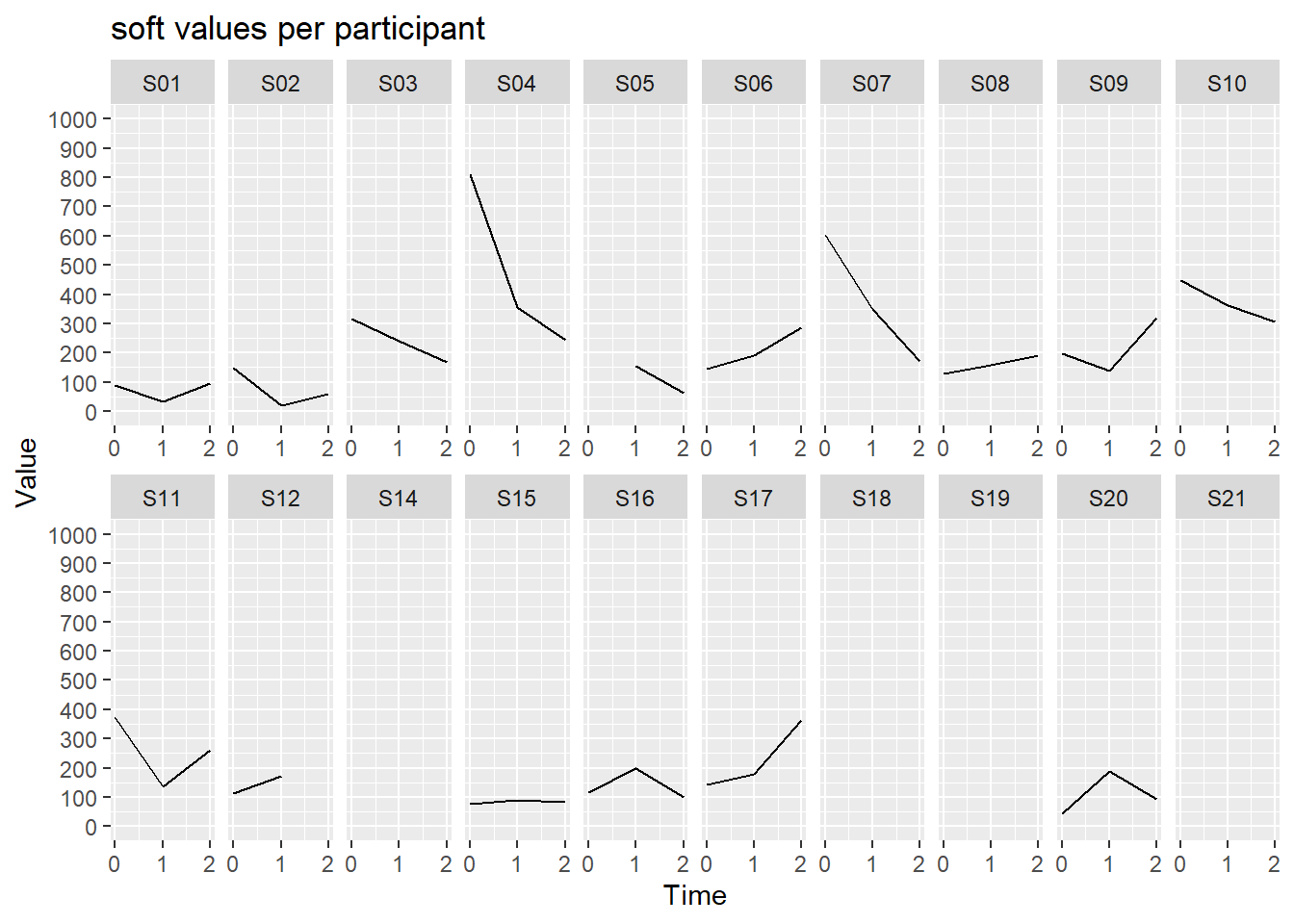
**

**
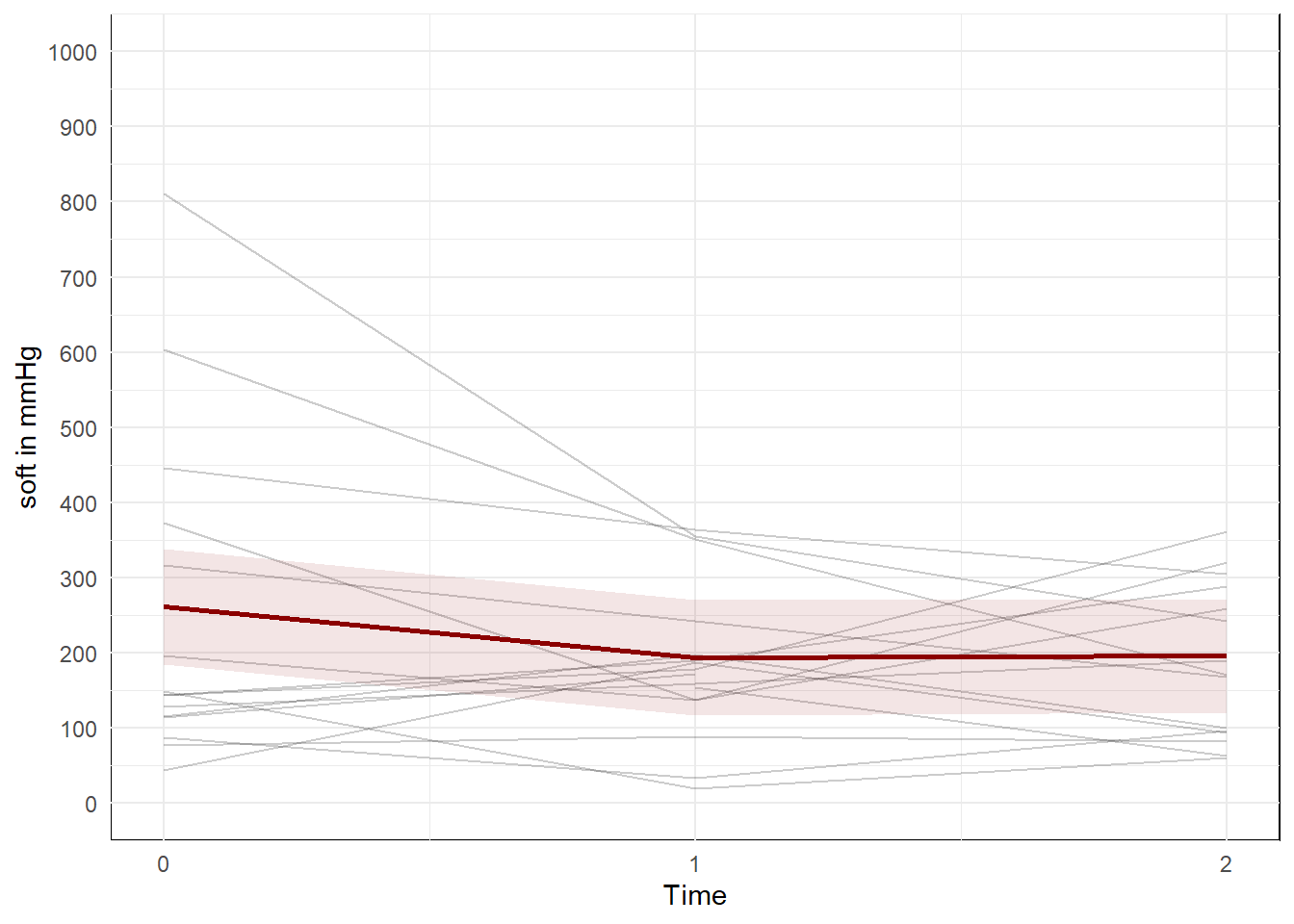
**

**
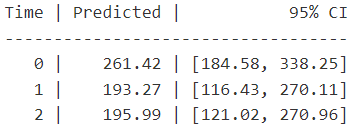
**

## Appendix 17: LME model on esophageal pressure (in mmHg) of a low sustained vowel /a/

**
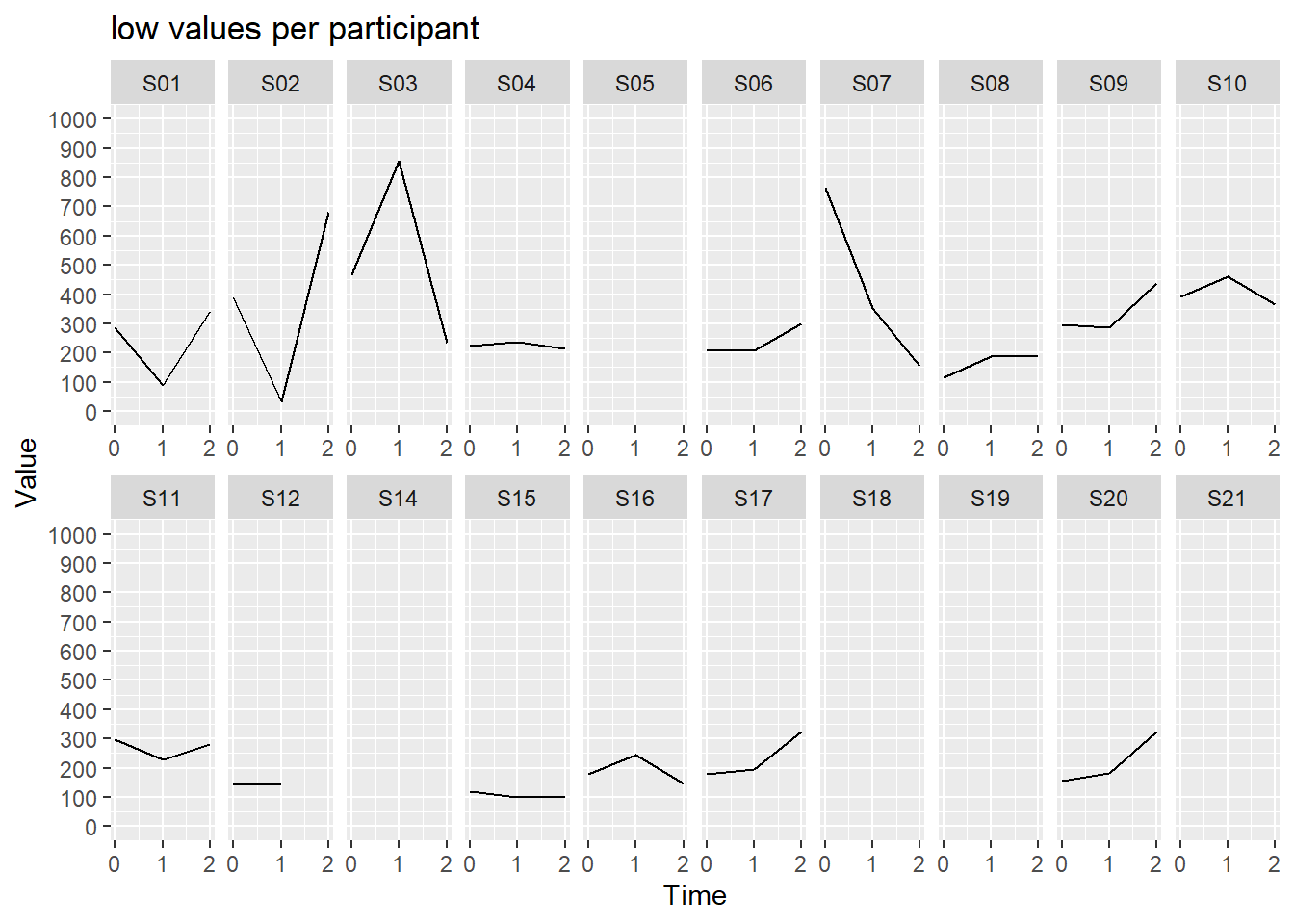
**

**
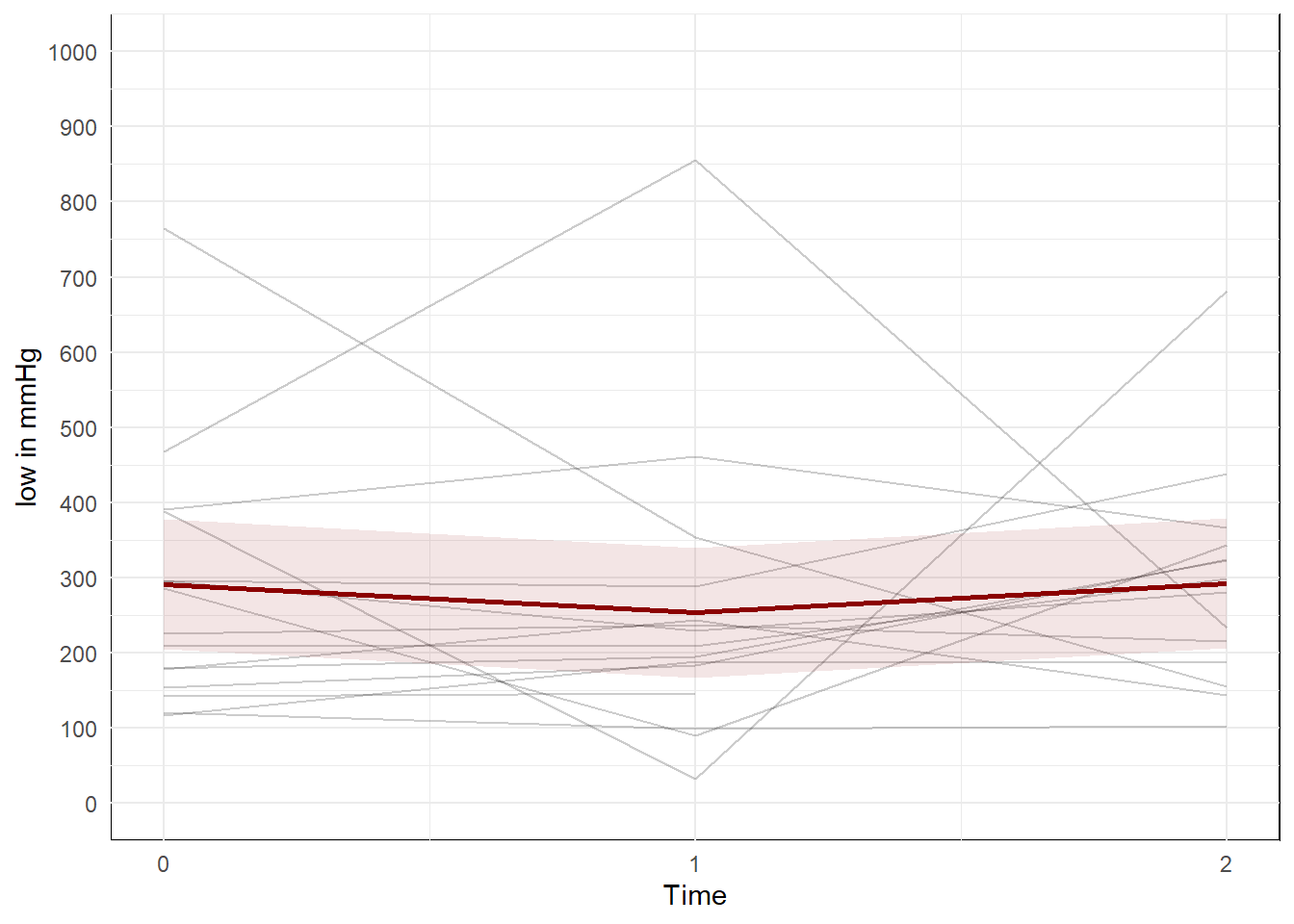
**

**
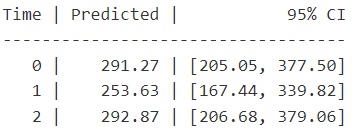
**

## Appendix 18: LME model on esophageal pressure (in mmHg) of a high sustained vowel /a/

**
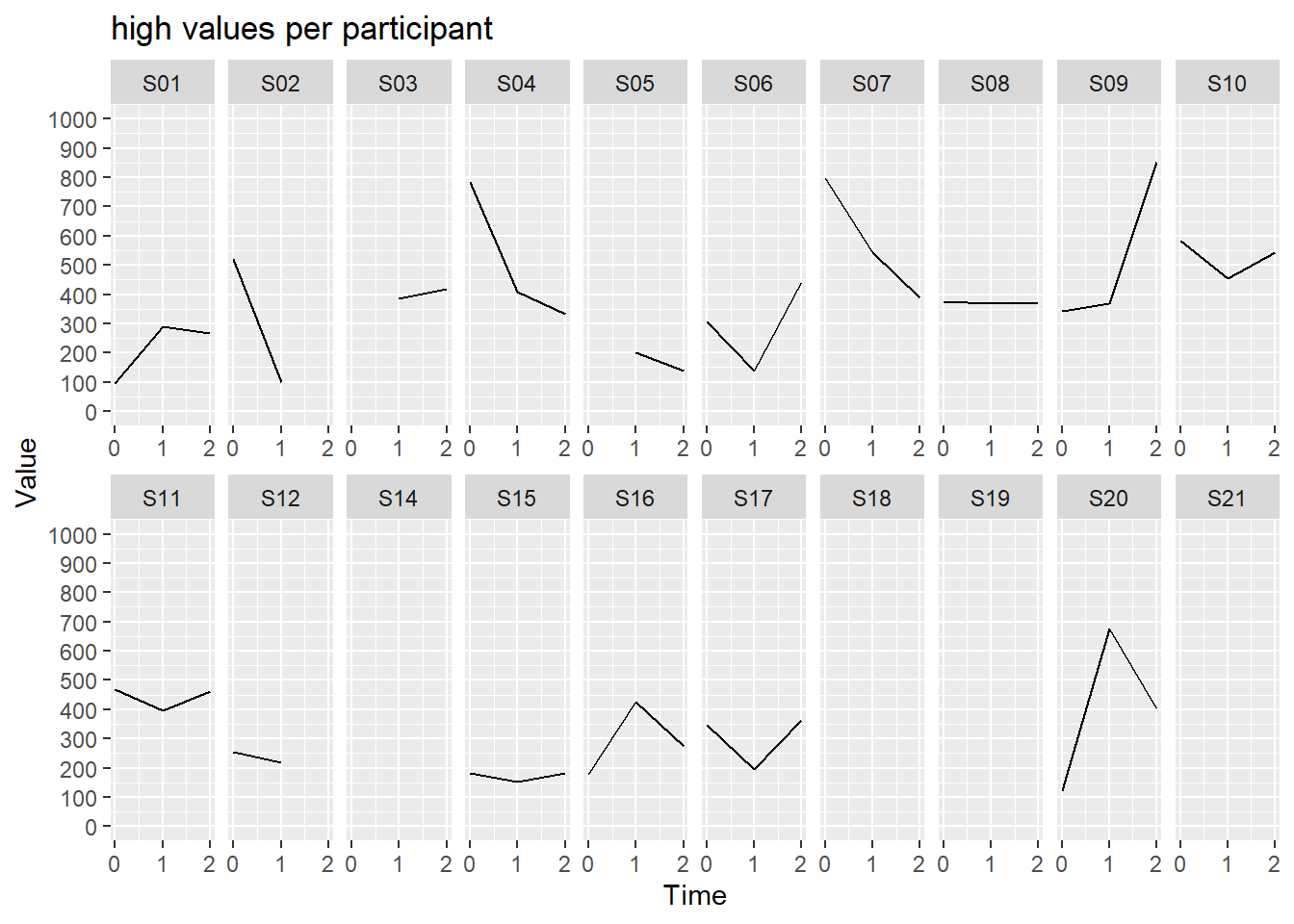
**

**
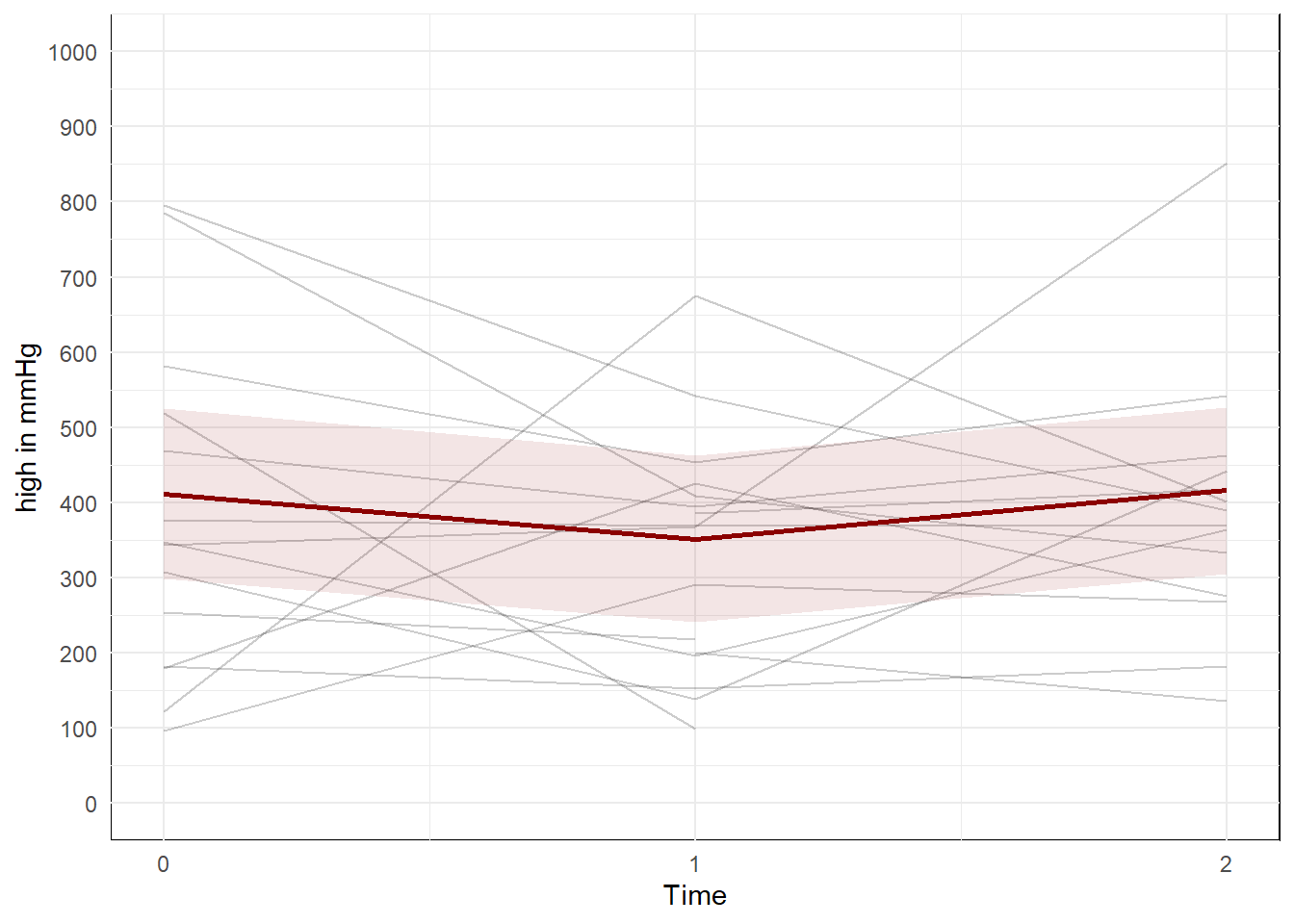
**

**
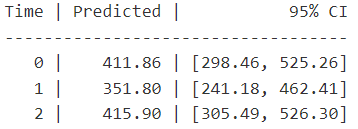
**

## Appendix 19: LME model on esophageal pressure (in mmHg) of a high sustained vowel /a/

**
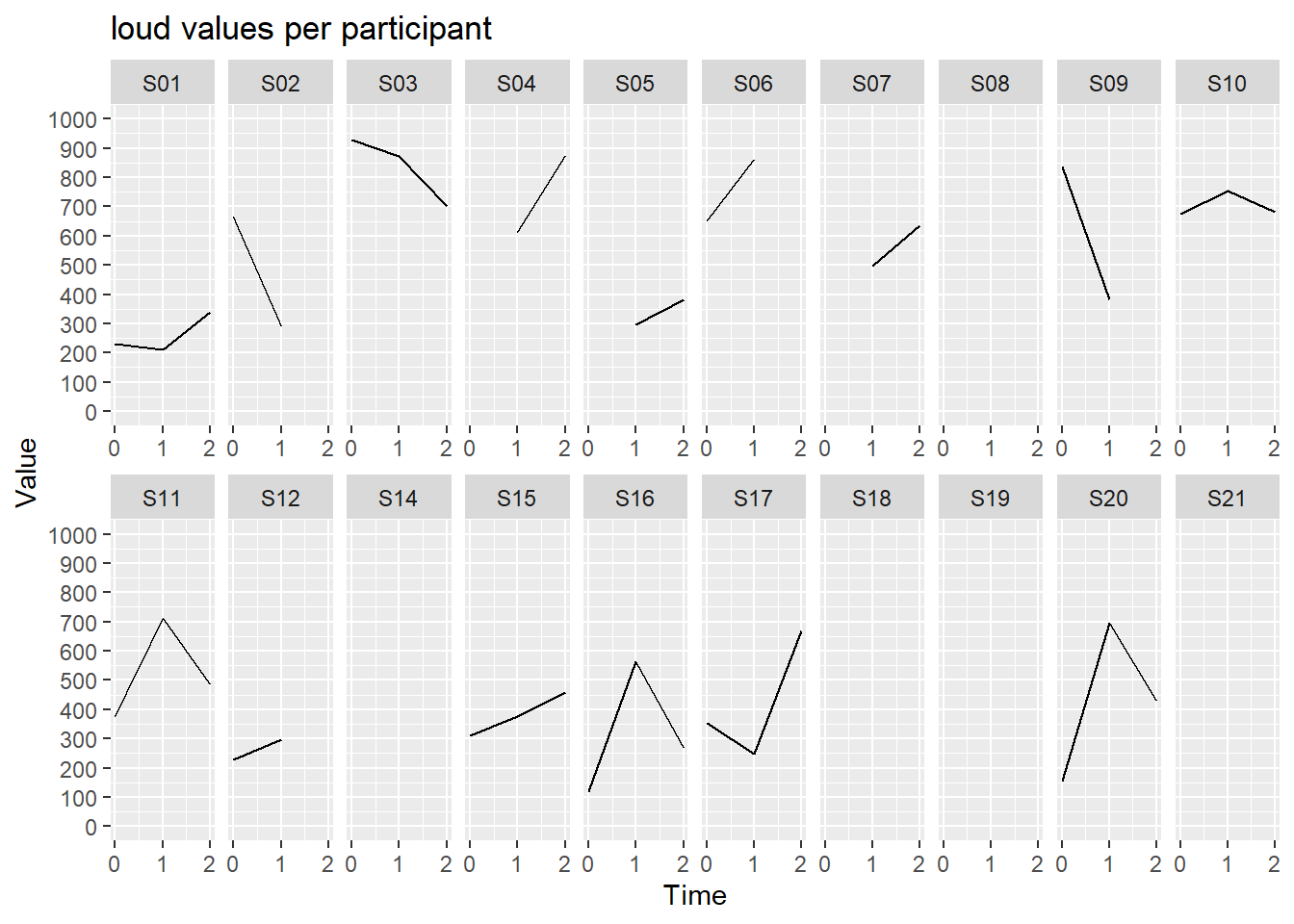
**

**
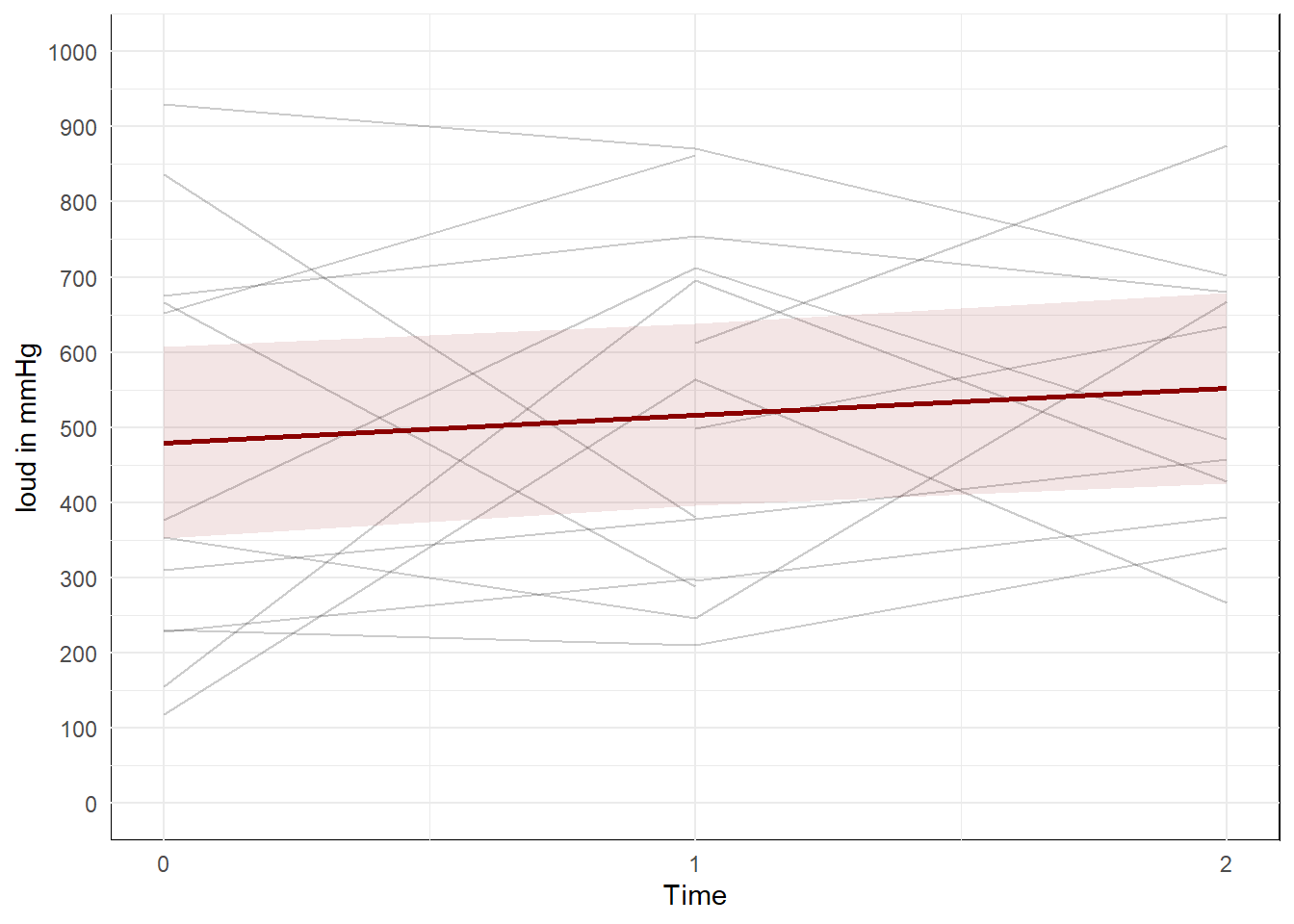
**


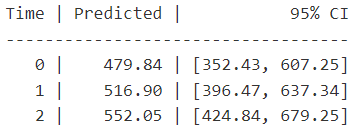

Supplement: Supplementary file 1 — Data S1.Supporting Information. [file HED-47-2209-s001.docx]
